# Phylogeny, biogeography and taxonomic re-assessment of *Multifurca* (Russulaceae, Russulales) using three-locus data

**DOI:** 10.1371/journal.pone.0205840

**Published:** 2018-11-07

**Authors:** Xiang-Hua Wang, Roy E. Halling, Valérie Hofstetter, Teresa Lebel, Bart Buyck

**Affiliations:** 1 Key Laboratory for Plant Diversity and Biogeography of East Asia, Kunming Institute of Botany, Chinese Academy of Sciences, Kunming, P. R. China; 2 New York Botanical Garden, Institute of Systematic Botany, Bronx, New York, United States of America; 3 Agroscope, Plant Protection, Mycology and Biotechnology Lab, Nyon, Switzerland; 4 National Herbarium of Victoria, Royal Botanic Gardens Victoria, Melbourne, Australia; 5 Institut de Systématique, Ecologie, Biodiversité (ISYEB), Muséum national D’histoire naturelle, CNRS, Sorbonne Université, Paris, France; Fred Hutchinson Cancer Research Center, UNITED STATES

## Abstract

*Multifurca* is a small genus newly established to accommodate lactarioid and russuloid species with some characters reminiscent of corticoid members of Russulaceae. It shows an amphi-pacific distribution with strong preference for the tropical zone of the Northern Hemisphere and thus has particular significance for biogeographical study. Using worldwide samples and three loci (ITS, 28S rDNA and *rpb*2), we demonstrated that *Multifurca* is split into two highly supported major clades that are here recognized at the subgeneric level: subg. *Furcata* subg. nov. exclusively includes lactarioid species, while subg. *Multifurca* includes species with a russuloid habit. Using phylogenetic species recognition and comparison of genetic distances we recognize five new and six previously described species, almost double the known number of species before this study. Molecular dating using a Bayesian method suggested that *Multifurca* originated in early Paleocene and diversified in the Eocene. The most recent interspecific divergences occurred both in Asia and America, roughly at the same time around the Pliocene. Ancestral area reconstruction and comparisons of genetic distances and morphology suggested an early divergence within Australasia or tropical Asia. From the early Miocene to Pliocene, multiple dispersals/migrations to Australasia and North America by island hopping or land bridge likely happened. Vicariance at the late Tertiary might be the most likely mechanism accounting for the eastern Asia—southeastern North America and Australasia—tropical Asia disjunct distributions. The shared polymorphisms in the ITS alignment, numerous degenerated base pairs in the *rpb2* sequences and weak conflict between the ITS and LSU genealogies of *M*. subg. *Furcata* suggest recent speciation. Host specificity of *Multifurca* species or species pairs is relatively low. Host shifts are believed to have aided establishment in new territories during the dispersals and migrations.

## Introduction

The traditional classification of Russulaceae, the core-group of the russuloid clade of Basidiomycota, has been revolutionized with the application of molecular data. Among the outcomes of this new approach, the definition of Russulaceae has been expanded to include corticiaceous taxa [1,2] while the traditional two agaricoid genera have been split into four genera [3], three of which still include secotioid-gasteroid and/or pleurotoid species [4–11]. This paper deals with the fourth and newly erected genus, *Multifurca* Buyck & V. Hofst., which has only six agaricoid species reported but has the peculiarity that it combines both russuloid and lactarioid species [3].

The type species of the genus, *M*. *ochricompacta* (Bills & O.K. Miller) Buyck & V. Hofst., was originally described from the eastern USA (Virginia) as a species of *Russula*. Because of its peculiar characters, it was placed in a new subsection, *R*. subsect. *Ochricompactae* Bills & O.K. Mill., of uncertain affinities with the remainder of the genus [12]. Besides *M*. *ochricompacta*, the other four species, i.e., *M*. *aurantiophylla* (Buyck & Ducousso) Buyck & V. Hofst., *M*. *furcata* (Coker) Buyck & V. Hofst., *M*. *stenophylla* (Berk.) T. Lebel et al. and *M*. *zonaria* (Buyck & Desjardin) Buyck & V. Hofst., were all originally described in either *Lactarius* or *Russula* [3, 12–17]. Although *M*. *roxburghiae* Buyck & V. Hofst. Buyck was originally described in *Multifurca*, its holotype was ever misidentified as “*Russula grossa*” [3]. Notwithstanding the fact that *Multifurca* contains both lactarioid and russuloid species, the genus is easily recognized by a combination of the following characters: at maturity yellow to salmon, narrow and regularly forked gills producing among the darkest spore prints in the family, a concentrically zonate context, unusually small spores and presence of an often strong, resinaceous or citronella smell that is unknown from other genera in Russulales. *Multifurca* is of particular phylogenetic importance for the phylogeny of Russulaceae as it is the only genus that exhibits features that are reminiscent of the earlier diversified resupinate genera *Gloeocystidiellum* Donk and *Boidinia* Stalpers & Hjortstam [3]. Although up to now there is no direct evidence showing that species of *Multifurca* are ectomycorrhizal, all the specimens were collected from ground in forests dominated by putative ectomycorrhizal hosts [3,12,14–19]. The possible mycorrhizal nature in contrast to a more or less resupinate (i.e. often saprotrophic) face makes it an intriguing group for biological study. Our belief that *Multifurca* might represent an ancient, relict lineage of Russulaceae is also supported by the rarity of its constituent species in their known distribution area [16,18,19]. With the exception of *M*. *stenophylla*, a species occurring in the cool temperate southern eucalypt and *Nothofagus* rain forests of south eastern Australia and Tasmania, the genus appears restricted to subtropical to tropical climates with known representatives in southern Asia, New Caledonia, southeastern USA and Central America [3,16–19]. Such an amphi-pacific distribution with strong preference for the tropics suggests an intriguing group for biogeographic analyses [20–23].

For the present paper, the authors have assembled worldwide collections of *Multifurca* in order to re-assess the morphological and molecular diversity of *Multifurca* using DNA sequence data of ITS, nuc 28S rDNA (28S) and part of the second largest subunit of the RNA polymerase II (*rpb*2) (the most frequently used three loci in Russulaceae), whereas molecular dating using a Bayesian method was used to trace the biogeographical history of the genus.

## Materials and methods

### Taxon and data sampling

Eighteen sequences published by Buyck et al. [3] and Lebel et al. [16] for *M*. *furcata*, *M*. *ochricompacta*, *M*. *stenophylla* and *M*. *zonaria* were retrieved from GenBank (Table 1). In addition, eleven *M*. *furcata*-like specimens, two of *M*. *zonaria* [19] and two of *M*. *roxburghiae* from central and southern China, three of *M*. *aurantiophylla* from New Caledonia, six of *M*. *furcata* and four of *M*. *ochricompacta* from central-southern and southeastern USA (Arkansas, Mississippi, Texas), and one of an undescribed species from eastern Australia were sampled to amplify the ITS region and 28S and *rpb2* genes. In the DNA dataset of Lebel et al. [16], only ITS and 28S were sequenced for *M*. *stenophylla* and the ITS sequences were incomplete. We amplified the complete ITS region and *rpb2* gene for four of the six specimens and one additional specimen of *M*. *stenophylla* collected from Tasmania, Australia (TL2462, MEL) to cover a broader geographical distribution.

**Table 1:** Samples used for molecular phylogenetic analyses of *Multifurca*.

species	voucher (herbarium)	origin	GenBank accession		
			ITS	28S	*rpb2*
*Lactarius pubescens*	U. Eberhardt 15.09.2002–2 (UPS)	Sweden	DQ421996	DQ421996	DQ421929
*Multifurca aurantiophylla*	B. Buyck et al. 09–023 (PC)	New Caledonia	**MH063874**	**MH063839**	**MH061171**
*M*. *aurantiophylla*	B. Buyck et al. 09–074 (PC)	New Caledonia	**MH063872**	**MH063837**	**MH061169**
*M*. *aurantiophylla*	B. Buyck et al. 09–345 (PC)	New Caledonia	**MH063873**	**MH063838**	**MH061170**
*M*. *aurantiophylla*	K18C (not specified)	New Caledonia	FJ656009	**—**	**—**
*M*. *australis*	R.E. Halling 10009 (BRI)	Queensland, Australia	**MH063871**	**MH063836**	**MH061168**
*M*. *furcata*	D.P. Lewis 4293 (FH)	Mississippi, USA	**MH063860** **MH063859**	**MH063828**	**MH061157**
*M*. *furcata*	D.P. Lewis 6495 (FH)	Mississippi, USA	**MH063863**	**MH063831**	**MH061160**
*M*. *furcata*	D.P. Lewis 6330 (FH)	Texas, USA	**MH063861**	**MH063829**	**MH061158**
*M*. *furcata*	D.P. Lewis 6743 (FH, epitype)	Texas, USA	**MH063862**	**MH063830**	**MH061159**
*M*. *furcata*	J. Justice 15.012 (PC)	Arkansas, USA	**MH063858**	**MH063827**	**MH061156**
*M*. *mesoamericana*	R.E. Halling 7804 (NY), holotype	Costa Rica	DQ421994	DQ421994	DQ421927
*M*. *mesoamericana*	R.E. Halling 8361 (NY)	Costa Rica	DQ421995	DQ421995	DQ421928
*M*. *ochricompacta*	B. Buyck 02.107 (PC)	Texas, USA	DQ421984	DQ421984	DQ421940
*M*. *ochricompacta*	J. Justice 2010.08 (PC)	Arkansas, USA	**MH063879**	**MH063844**	**MH061176**
*M*. *ochricompacta*	B. Buyck & V. Hoftetter 14–013 (PC)	Texas, USA	**MH063877**	**MH063842**	**MH061174**
*M*. *ochricompacta*	B. Buyck & V. Hoftetter 14–001 (PC)	Texas, USA	**MH063878**	**MH063843**	**MH061175**
*M*. *orientalis*	F. Li 1055 (KUN)	Guangdong, China	**MH063857**	**MH063826**	**MH061155**
*M*. *orientalis*	X.H. Wang 3034 (KUN), holotype	Anhui, China	**MH063856**	**MH063825**	**MH061154**
*M*. *pseudofurcata*	Q. Cai 525 (KUN)	Yunnan, China	**MH063855**	**MH063824**	**MH061153**
*M*. *pseudofurcata*	J. Li 61 (KUN)	Yunnan, China	**MH063853**	**MH063822**	**MH061151**
*M*. *pseudofurcata*	X.H. Wang 2374 (KUN)	Guizhou, China	**MH063852**	**MH063821**	**MH061150**
*M*. *pseudofurcata*	B. Xu s.n. (HKAS 52928, KUN)	Sichuan, China	**MH063854**	**MH063823**	**MH061152**
*M*. *pseudofurcata*	X.H. Wang 2844 (KUN)	Yunnan, China	**MH063851** **MH063850**	**MH063820**	**MH061149**
*M*. *pseudofurcata*	G. Wu 85 (KUN)	Yunnan, China	**MH063847**	**MH063817**	**MH061146**
*M*. *pseudofurcata*	L.P. Tang 1093 (KUN)	Yunnan, China	**MH063846**	**MH063816**	**MH061145**
*M*. *pseudofurcata*	X.H. Wang 3205 (KUN), holotype	Yunnan, China	**MH063849**	**MH063819**	**MH061148**
*M*. *pseudofurcata*	R. Wang 2011-yl-69 (KUN)	Yunnan, China	**MH063848**	**MH063818**	**MH061147**
*M*. *pseudofurcata*	xp2-20120922-01 (KUN)	China	**KR364125**	**—**	**—**
*M*. *roxburghiae*	X.H. Wang 3650 (KUN)	Henan, China	**MH063875**	**MH063840**	**MH061172**
*M*. *roxburghiae*	X.H. Wang 669 (KUN)	Yunnan, China	**MH063876**	**MH063841**	**MH061173**
*Multifurca* sp	2676 (not specified)	Malaysia	KP071201	**—**	**—**
*M*. *stenophylla*	T. Lebel 2335 (MEL), epitype	Tasmania, Australia	**MH063867**	JX266636	**MH061164**
*M*. *stenophylla*	T. Lebel & P. Catcheside TL2462 (MEL)	Tasmania, Australia	**MH063864**	**MH063832**	**MH061161**
*M*. *stenophylla*	C. Dunk 584 (MEL)	Victoria, Australia	**MH063868**	JX266633	**MH061165**
*M*. *stenophylla*	C. Dunk 600 (MEL)	Victoria, Australia	**MH063866**	**MH063833**	**MH061163**
*M*. *stenophylla*	J.E. Tonkin 1201 (MEL)	Victoria, Australia	**MH063865**	JX266634	**MH061162**
*M*. *zonaria*	D.E. Desjardin7442 (SFSU), holotype	Thailand	DQ421990	DQ421990	DQ421942
*M*. *zonaria*	A. Verbeken 2004–032 (GENT)	Thailand	DQ422000	DQ422000	DQ421947
*M*. *zonaria*	X.H. Wang 1790 (KUN)	Yunnan, China	**MH063870**	**MH063835**	**MH061167**
*M*. *zonaria*	X.H. Wang 1984 (KUN)	Yunnan, China	**MH063869**	**MH063834**	**MH061166**

Sequences produced in the present study are in bold. Two ITS copies were obtained from XHW2844 and DPL4293 by cloning respectively.

The samples/data used in this study covered all previously described species of *Multifurca* and included five holotypes (*M*. *orientalis*, *M*. *pseudofurcata*, *M*. *zonaria* and two new species described below) and two epitypes (*M*. *furcata* and *M*. *stenophylla*). In addition, we retrieved three ITS sequences from GenBank by BLASTn: KP071201 labeled as *R*. *japonica* (from a Malaysian sample), FJ656009 from a New Caledonian sporocarp sample by Prin et al. (unpublished) and KR364125 from a Chinese sample labelled as *Multifurca* sp. [24]. These three sequences were included in the ITS dataset.

To test if the monophyly of *Multifurca* could still be retrieved after adding newly introduced taxa, seven species of *Lactarius*, 11 of *Lactifluus* and eight of *Russula* were added to construct a phylogeny. These taxa represented the various subgenera of these three genera based on the phylogeny of Russulaceae and DNA data published in former literatures [11,24,25] (S1 Fig). After testing monophyly of the genus, we reduced the outgroup sampling to infer internal relationships within *Multifurca*.

### DNA extraction, PCR amplifications and sequencing

For nine newly collected Chinese samples (less than ten years old), DNA extraction and PCR protocols followed Wang et al. [26]. For the other samples, DNA was extracted using a CTAB protocol [27] and purified with GeneClean® II Kit (MP Biomedicals), following the manufacturer’s instruction. The complete ITS region of the sample XHW2844 could not be successfully obtained by direct sequencing. For this sample, PCR product was cloned using the Takara® pMD^TM^18T cloning kit (Dalian, China) following the manufacturer’s instruction. DNA insert of positive bacterial colonies was amplified and sequenced using the primer pair of the vector (M13F+M13R). Four clones with the desired length of PCR product were sequenced. Sequences of clones differing only in base substitutions were merged into one sequence by replacing the substitutions with degenerate bases; for clones with different INDELs, we present different sequences with these INDELs under the same sample (Table 1). The same cloning method was used to phase the heterozygosity of the ITS2 region of DPL4293 and to obtain sequences with weak PCR products.

General primers for ITS (ITS1F+ITS4, ITS5+ITS4) [28], 28S (LROR+LR5) [29,30] and *rpb2* (RPB2-6F+fRPB2-7cR) [31] were mostly used to amplify the ITS, 28S and *rpb2* regions of the newly collected specimens. For some collections (18–29 years old), a batch of internal primers were designed to obtain the three loci, based on the alignments of the obtained sequences. These primers are listed and shown in S1 Table. Raw sequences were assembled with Sequencher v4.1.4 (Gene Codes Corporation) and sequences are deposited in GenBank (Table 1).

### Alignments and molecular phylogenetic analyses

Alignments were made using the online version of the multiple sequence alignment program MAFFT v7 [32], applying the L-INS-I strategy and manually adjusted in BioEdit [33]. To test if potential ambiguous sites in the ITS alignment would add noise to the phylogenetic analyses, we used different sets of parameters, from conservative to tolerant to select sites in Gblocks v0.91b [34] and compared the generated Maximum Likelihood (ML) trees and the ML Bootstrap Proportions (ML-BP) from each parameter set with the 28S and *rpb2* trees and corresponding ML-BP. When testing the reciprocal monophyletic relationships of *Multifurca*, *Lactarius*, *Lactifluus* and *Russula*, only 28S and exons of *rpb2* were used. For the subsequent analyses, we kept the alignable intron of *rpb2* and used the three loci. In the concatenated ITS-28S-*rpb2* dataset, we used duplicate sequences of 28S and *rpb2* for the ITS copies of XHW2844 and DPL4293 respectively and removed the GenBank-retrieved sample xp2-20120922-01 of *M*. *pseudofurcata* because it has only ITS sequence (KR364125) that is identical with some of our sequences. The ITS, 28S and *rpb2* alignments are deposited in TreeBASE as S21024.

To test for combinability between the ITS, 28S and *rpb2* genealogies, we conducted ML bootstrap and Bayesian analyses on individual datasets sampling only the taxa for which the three loci were available. Phylogenetic conflict was assumed to be significant when two different relationships (one monophyletic and the other non-monophyletic) for the same set of taxa were both supported with ML-BP ≥ 70% and posterior probability of Bayesian Inference (BI-PP) ≥ 95%.

Phylogenetic analyses were conducted using ML in RAxML v7.2x [35] and BI in MrBayes v3.2.6 [36]. The dataset *rpb2* was partitioned as follows: first and second codon positions, third codon position and intron. The ITS-28S-*rpb2* combined dataset was partitioned as follows: ITS, 28S and the above three partitions for *rpb2*. ML analyses were conducted applying the Rapid Bootstrapping algorithm with 1000 replicates, followed by an ML tree search. For BI analyses, the best-fit models were selected using MrModeltest [37]. The BI analyses were conducted using four runs with four chains each for one million generations sampling one tree every 100^th^ tree. Runs were inspected to make sure the average standard deviation of split frequencies went below 0.01 and effective sampling sizes were > 200 in Tracer v1.7.0 [38]. A 50% majority rule consensus tree was built after discarding trees from the burn-in (first 25% of the trees). Trees generated by the two analyses were viewed and exported in FigTree v1.3.1. Trees were rooted with *Lactarius pubescens*.

### Species delimitation using DNA data

Genealogical Concordance Phylogenetic Species Recognition (GCPSR) [39,40] was used to recognize the terminal independent evolutionary lineages, using only samples for which all three loci were obtained. A cluster of individuals was taken as an independent evolutionary lineage if it met with one of the two following criteria: i) the clade was present in at least two of the three ML genealogies; ii) its monophyly was highly supported by both ML-BP ≥70% and BI-PP ≥0.95 in at least one of the three genealogies and was not contradicted in any of the other genealogies at the same level of support. Exhaustive subdivision was then used for deciding which independent evolutionary lineages represented phylogenetic species [40]. That is to say, in the combined tree of the three loci, if any individual was not included in one of the terminal evolutionary lineages, we traced down the nodes of the tree from that individual until all individuals were included in evolutionary lineages.

To determine if the singletons “2676” and “RH10009” (that GCPSR does not fit) represent real species, we calculated two sets of genetic distances: within-group mean distances of all the species recognized by GCPSR and between-group mean distances between those two singletons and the other species. If the lowest between-group mean distance was bigger than the highest within-species distance, we separated them into two species, otherwise we merged them into their sister species. For “2676”, we used ITS data and for RH10009 the combined ITS-28S-*rpb2* data to calculate mean distances in MEGA5 [41], using the following settings: Bootstrap method (1000 replicates) as the variance estimation method, Maximum Composite Likelihood model as the substitution model, Gamma distribution as the rates among sites, different patterns among lineages, and pairwise deletion for gaps treatment.

### Biogeographical analysis

We followed with minor modifications the methods of Dentinger et al. [42] and Feng et al. [43] to estimate the divergence times and infer the center of origin of *Multifurca*. Specifically, we used 28S and *rpb2* datasets composed of 26 *Multifurca* and 36 outgroup taxa (S2 Table). *Rhizopus oryzae* Went & Prins. Geerl. (Mucoromycotina) was used to set the time of the most recent common ancestor (tMRCA) of Ascomycota and Basidiomycota, four species to represent the major clades of Ascomycota and 31 species representative of the major clades of Basidiomycota [44,45]. We included *Bondarzewia montana* (Quél.) Singer, *Echinodontium tinctorium* (Ellis & Everh.) Ellis & Everh., *Stereum hirsutum* (Willd.) Pers., *Gloeopeniophorella convolvens* (P. Karst.) Boidin et al., four species of *Russula*, three of *Lactarius* and four of *Lactifluus* to cover the genetic diversification of the russuloid clade and the sibling genera of *Multifurca* [1,11,24,44,46]. The 26 samples of *Multifurca* were selected to cover the genetic differentiation of the genus as displayed by the ITS, 28S and *rpb2* sequences. In order to refer to the geological times of *Mycena*-like and *Rhizopogon*-*Suillus*-like fossils [47,48], we included *Mycena aurantiidisca* (Murrill) Murrill, *M*. *galericulata* (Scop.) Gray, *Rhizopogon nigrescens* Coker & Couch and *Suillus spraguei* (Berk. & M.A. Curtis) Kuntze [formerly as *S*. *pictus* (Peck) Kuntze] to represent the possible phylogenetic position of *Mycena* and the suilloid clade [49,50].

For the *rpb2* alignment, homogeneity of translated proteins was referred to improve the nucleotide alignment. The only intron of *rpb2* was excluded from analyses. BEAST 1.8.0 [51] was used to estimate divergence times. To produce the executable xml file by BEAUTi (implemented in BEAST), we set parameters as follows: 28S and *rpb2* datasets were set as two partitions, with substitution and molecular clock models unlinked and trees linked; taxa were constrained to monophyletic sets by referring to the SSU+28S+*rpb2* or nrDNA+*rpb2*+*ef1a* phylogenies of fungi [44, 52], the multi-gene phylogenies of *Lactarius* [46], *Lactifluus* [24] and *Russula* [11] and our three-locus phylogeny of *Multifurca;* Following the outcomes of model selection, GTR+I+G model was used as the substitution and site heterogeneity model and best frequencies were set as estimated; a lognormal relaxed clock was used for molecular clock analysis; Yule process was used as speciation for tree priors; ucld.mean of the 28S and *rpb2* datasets were set to 0.033, with upper value = 1.0 and lower value = 0; for fossil calibration, we set mean = 575 Ma with a standard deviation of 38.26 Ma for the tMRCA of Ascomycota and Basidiomycota, inferred from the estimated time range of 500–650 Ma of the stem base of Ascomycota [53] based on the well-preserved ascomycetes fossil *Paleopyrenomycites* [54,55]. The reason why we selected this more ancient time origin instead of the more recent one of 452 Ma inferred by previous studies [56,57] is that the distribution of *Multifurca* in the South Pacific seems to suggest a Gondwana affinity and an earlier origin will be in favor of testing such an assumption. Two independent runs were conducted in BEAST for 20 million generations. Logfiles of the runs were inspected in Tracer v1.7.0 [38], to monitor if the ESS values of all parameters exceeded 200. Logfiles of the two runs were combined in Tracer v.1.7.0 by setting 10% logs as the burn-in. Tree files of the two runs were combined by in LogCombiner v1.8.0 [51] by setting 10% as the burn-in and processed in TreeAnnotator v1.8.0 [51] and then viewed and exported in FigTree 1.3.1.

To infer the center of origin for *Multifurca* and its species, we selected *Multifurca* from the chronogram generated by BEAST above for further analysis. We used ML analysis and Dispersal-Extinction-Cladogenesis (DEC) model implemented in LAGRANGE [58] provided in RASP v3.2 [59] to do the ancestral area reconstruction (AAR). The geographical areas of the extant species were defined as three regions: Australasia (A), Mainland and southeast Asia (B), and North and Central America (C). In LAGRANGE, for range constraints, we set the maximum areas as two and excluded the constrained area AC considering the two individual regions have never been adjacent. Dispersal probabilities between different areas were constrained following Clayton et al. [60], except that for the period 0–5 Ma we set the dispersal probabilities between Asia and America as zero, considering the cold climate at that time and the thermophilic habit of *Multifurca* species.

### Morphology

Descriptions of sporocarps were from fresh material, and micro-morphological study was performed on dried material. Basidiospores were observed and measured in Melzer’s reagent in side view, excluding ornamentation and apiculus. All other micro-morphological structures were revived in 5% KOH then mounted with Congo-red (aqueous reagent). All drawings except those of the basidiospores were made with the aid of a drawing tube installed on a Nikon E400 microscope. Colour codes refer to Kornerup & Wanscher [61] and abbreviations of herbaria follow Index Herbariorum. Measurements of spores were given as follows: (MIN) mean–SD–***mean****–*mean+SD (MAX), and this for length, width and Q ratio; SD is standard deviation, while MIN or MAX is the absolute minimum, resp. maximum value measured. These values were only added (in between brackets) if they were lower, resp. higher, than the mean values minus, resp. plus, standard deviation.

### Nomenclature

The electronic version of this article in Portable Document Format (PDF) in a work with an ISSN or ISBN will represent a published work according to the International Code of Nomenclature for algae, fungi, and plants, and hence the new names contained in the electronic publication of a PLOS article are effectively published under that Code from the electronic edition alone, so there is no longer any need to provide printed copies.

In addition, new names contained in this work have been submitted to MycoBank from where they will be made available to the Global Names Index. The unique MycoBank number can be resolved and the associated information viewed through any standard web browser by searching the MycoBank numbers contained in this publication to the website http://www.mycobank.org. The online version of this work is archived and available from the following digital repositories: PubMed Central and LOCKSS.

## Results

### DNA alignments and characters

**Fig 1:**
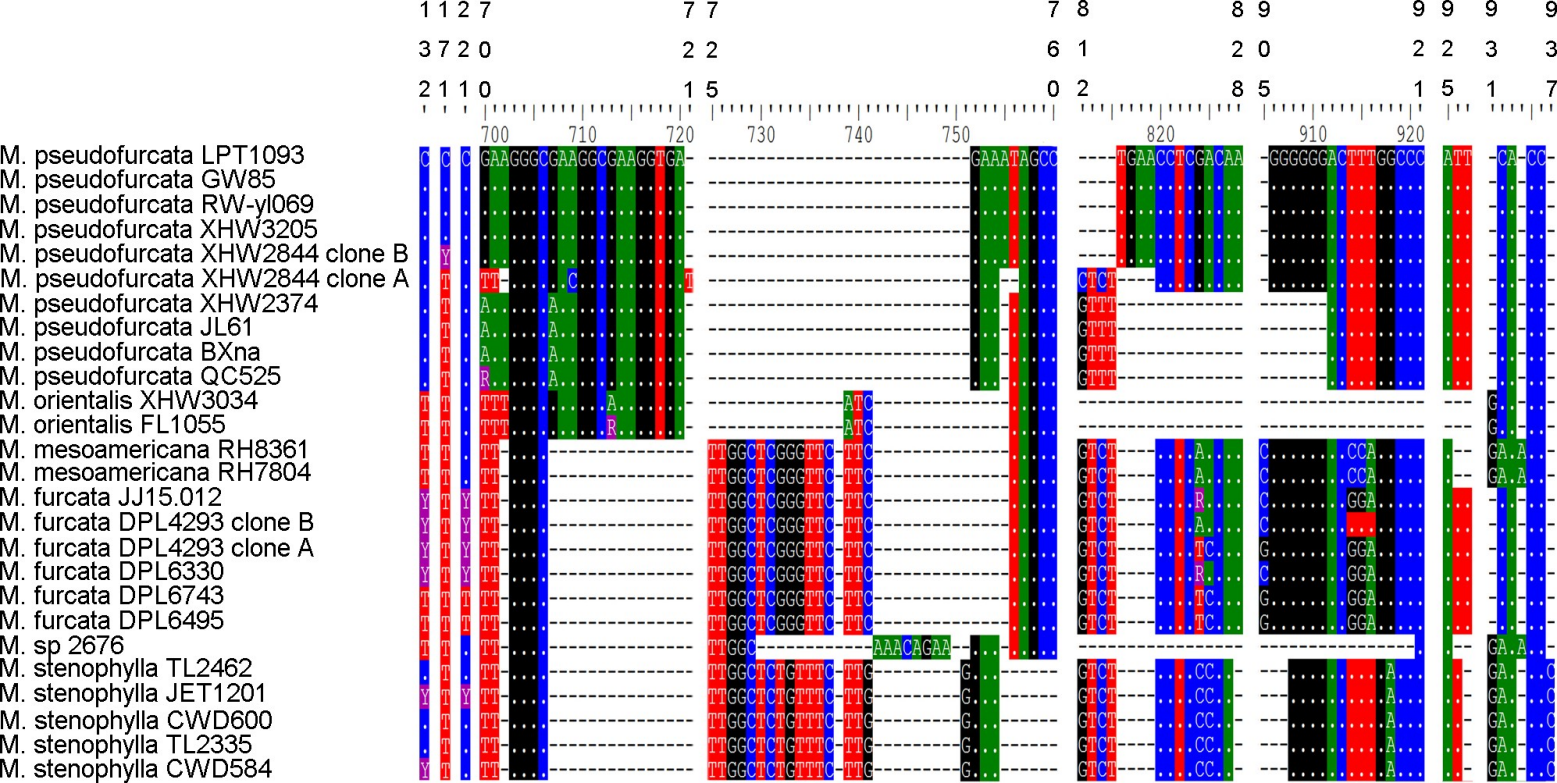
Selected sites of the ITS alignment of lactarioid samples of *Multifurca*.

The aligned ITS dataset includes 43 sequences and 938 characters: 345 bp of ITS1, 155 bp of 5.8S, and 438 bp of ITS2. The two ITS copies (GenBank accessions MH063850 and MH063851) of XHW2844 differ in six INDELs (Fig 1). Three of the six INDELs (sites: 700–701, 702, 816–819) are identical with those of *M*. *mesoamericana* and *M*. *stenophylla*, one (sites: 812–815) highly homogeneous with those of *M*. *furcata* and *M*. *stenophylla* and two (sites: 721, 755–756) unique. One of the two ITS copies of DPL4293 (GenBank accession MH063859) is homogeneous with that of *M*. *mesoamericana* at site 824 and with *M*. *pseudofurcata* and *M*. *stenophylla* at sites 914–916 and the other copy (MH063860) is the same as those of two samples of *M*. *furcata* (DPL6743 and DPL6495) (Fig 1). In addition, all taxa of the lactarioid clade more or less share INDELs with at least one of the other species (Fig 1).

In comparison with the ITS dataset, the 28S and *rpb2* alignments lack three samples, *Multifurca* sp 2676, K18C of *M*. *aurantiophylla* and xp2-20120922-01 of *M*. *pseudofurcata* due to unavailability of the other two loci (Table 1). All the samples of *Multifurca* have the same length of intron in *rpb2*, except that LPT1093 (*M*. *pseudofurcata*) is three base pairs shorter. Heterozygous loci in the *rpb2* dataset are much more common than in the ITS and 28S datasets: among the 202 polymorphic sites of *Multifurca*, 69 sites are degenerated.

### Phylogenetic relationships

The three ITS datasets with sites selected by conservative to tolerant parameters in Gblocks produced similar topologies (trees not shown). The dataset produced by the most conservative setting gave less resolved topology among the samples of *M*. *furcata*, *M*. *mesoamericana*, *M*. *orientalis* and *M*. *pseudofurcata* and among *M*. *aurantiophylla*, *M*. *australis* and *M*. *zonaria* and produced the lowest support values on nearly all the branches. In contrast, the support values produced by the dataset selected by the tolerant setting and un-Gblocked dataset are more comparable with that by the 28S and *rpb2* datasets. It seems that the ambiguous sites in the ITS alignment did not cause much noise in the phylogeny and based on this we decided to use the original ITS dataset for further analyses.

**Fig 2:**
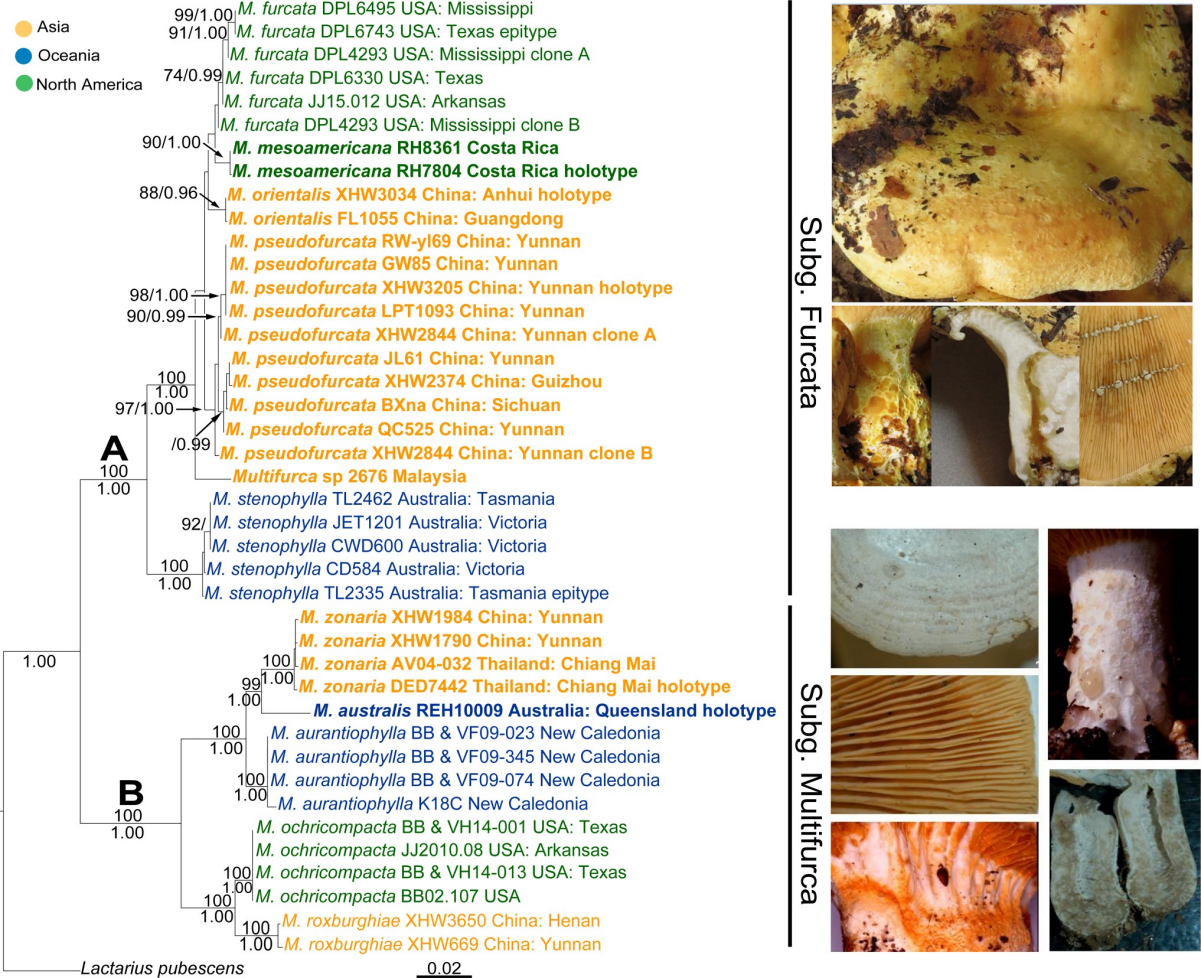
Maximum Likelihood (ML) phylogram of *Multifurca* based on the ITS-28S-*rpb2* combined dataset, rooted with *Lactarius pubescens*. Bootstrap proportions higher than 70% in the ML analysis (ML-BP) and posterior probabilities of the Bayesian Inference (BI-PP) higher than 95% are indicated above and below the branches respectively or as ML-BP/BI-PP by the node. New species are in bold. Initials proceeding sample numbers refer to the collectors (see Table 1).

MrModeltest selected HKY+G, GTR+I+G, SYM+G and SYM+G as the best-fit models for the ITS, 28S, *rpb2* and ITS-28S-*rpb2* datasets for the BI analyses. ML and BI analyses of the respective ITS, 28S and *rpb2* datasets produced almost identical topologies with comparable support values. The ITS, 28S and *rpb2* phylogenetic analyses inferred all identical deep and middle rank relationships but their respective topology solved different terminal relationships for *M*. *furcata*, *M*. *mesoamericana*, *M*. *orientalis* and *M*. *pseudofurcata* (refer to S2 Fig). However no significant conflict was detected between the topologies inferred by individual locus. The ML and BI analyses of the three-locus combined dataset produced identical topologies with comparable support values (Fig 2).

Analyses of the concatenated dataset produced a highly resolved phylogeny, with two major clades supported with ML-BP 100% and BI-PP 1.00 (A and B, Fig 2). The two major clades are represented by latex producing species (clade A) and species lacking latex (clade B) respectively. In clade A, samples of the Australian species *M*. *stenophylla* formed a basal clade, sister to the clade formed by all the Asian and American samples with an “*M*. *furcata*” morphology. Combined analyses of the ITS-28S-*rpb2* dataset did not resolve relationships among *M*. *furcata*, *M*. *mesoamericana*, *M*. *orientalis*, *M*. *pseudofurcata* and *Multifurca* sp (refer to S2 Fig) better than single locus analyses. In clade B, *M*. *zonaria* is the only species with hymenophoral pseudocystidia and yellowish pileus and *M*. *aurantiophylla* is the only one with well-differentiated pileocystidia. Eastern Australian *M*. *australis*, represented by a singleton on a long branch, was sister to *M*. *zonaria* with moderate to high support in the 28S and *rpb2* genealogies, as well as in the three-locus phylogeny, where they formed a clade sister to that formed by the North American *M*. *ochricompacta* and Asian *M*. *roxburghiae*. Compared to the species of clade A, relationships among all the species of clade B are well resolved. Distance analysis on the ITS-28S-*rpb*2 combined dataset showed that the within-group mean distance of clade B (0.035 ± 0.003) is almost double that of clade A (0.018 ± 0.002).

### Species delimitation

Using the two criteria for determining evolutionary lineages in GCPSR, i.e. genealogical concordance and/or genealogical non-disconcordance [40], ten terminal independent evolutionary lineages were recognized. The tenth lineage, only supported by ITS analyses (ML-BP/BI-PP 98/1.00), comprised four out of the eight *M*. *pseudofurcata* sampled in this study (S2 Fig: group 2 [QC525, BXna, JL61 and XHW2374]). The split of *M*. *pseudofurcata* in two independent lineages is not significantly conflicting with 28S or *rpb*2 genealogies (S2 Fig). By exhaustive subdivision, DPL6330 and PC0723659 were merged into the lineage of DPL6495 and DPL6743 to form *M*. *furcata*. As an outcome of GCPSR, ten phylogenetic species (taxa) were recognized (Table 2, indicated by stars in S2 Fig).

**Table 2:** Recognized phylogenetic species and corresponding support values from individual genealogies and from the three-locus combined phylogeny.

species recognized	ITS	28S	*rpb2*	three-locus combined	criterion applied
*M*. *aurantiophylla*	100/1.00	–/–	100/1.00	100/1.00	a and b
*M*. *furcata*	67/0.73	64/0.93	–/–	71/1.00	a
*M*. *mesoamericana*	99/0.99	100/1.00	–/–	100/1.00	a and b
*M*. *ochricompacta*	95/0.72	97/0.83	100/1.00	100/1.00	a and b
*M*. *orientalis*	–/0.66	100/0.99	98/1.00	100/1.00	a and b
*M*. *pseudofurcata* group 1	96/0.78	95/0.99	–/–	100/1.00	a and b
*M*. *pseudofurcata* group 2	95/0.99	–/–	–/–	–/–	b
*M*. *roxburghiae*	100/1.00	94/1.00	100/1.00	100/1.00	a and b
*M*. *stenophylla*	99/0.99	100/1.00	100/1.00	100/1.00	a and b
*M*. *zonaria*	68/0.97	83/0.96	100/1.00	100/1.00	a and b

For criterion applied, a: genealogical concordance, b: genealogical non-disconcordance. Limits of these species correspond to the stars in S2 Fig. Values are shown as ML-BP/BI-PP. Missing values apply to non-monophyly.

For the ITS dataset (S1 Text), the highest within-group mean distance (0.005) was for *M*. *aurantiophylla*. The lowest between-group value for the Malaysian sample 2676 (ITS: KP071201) is 0.022 (between *M*. *mesoamericana*) and based on this, we took it as a distinct species (*Multifurca* sp). For the ITS-28S-*rpb2* dataset (S1 Text), the highest within-group mean distance (0.003) is for *M*. *pseudofurcata* group 2. The lowest between-group value for the Australian sample RH10009 is 0.037 (between *M*. *zonaria*) and based on this, we recognized it as another species (*M*. *australis*). In total, we delimitated twelve putative species in *Multifurca* using DNA data, five in *M*. subg. *Multifurca* and seven in *M*. subg. *Furcata*.

### Divergence time estimation

**Fig 3:**
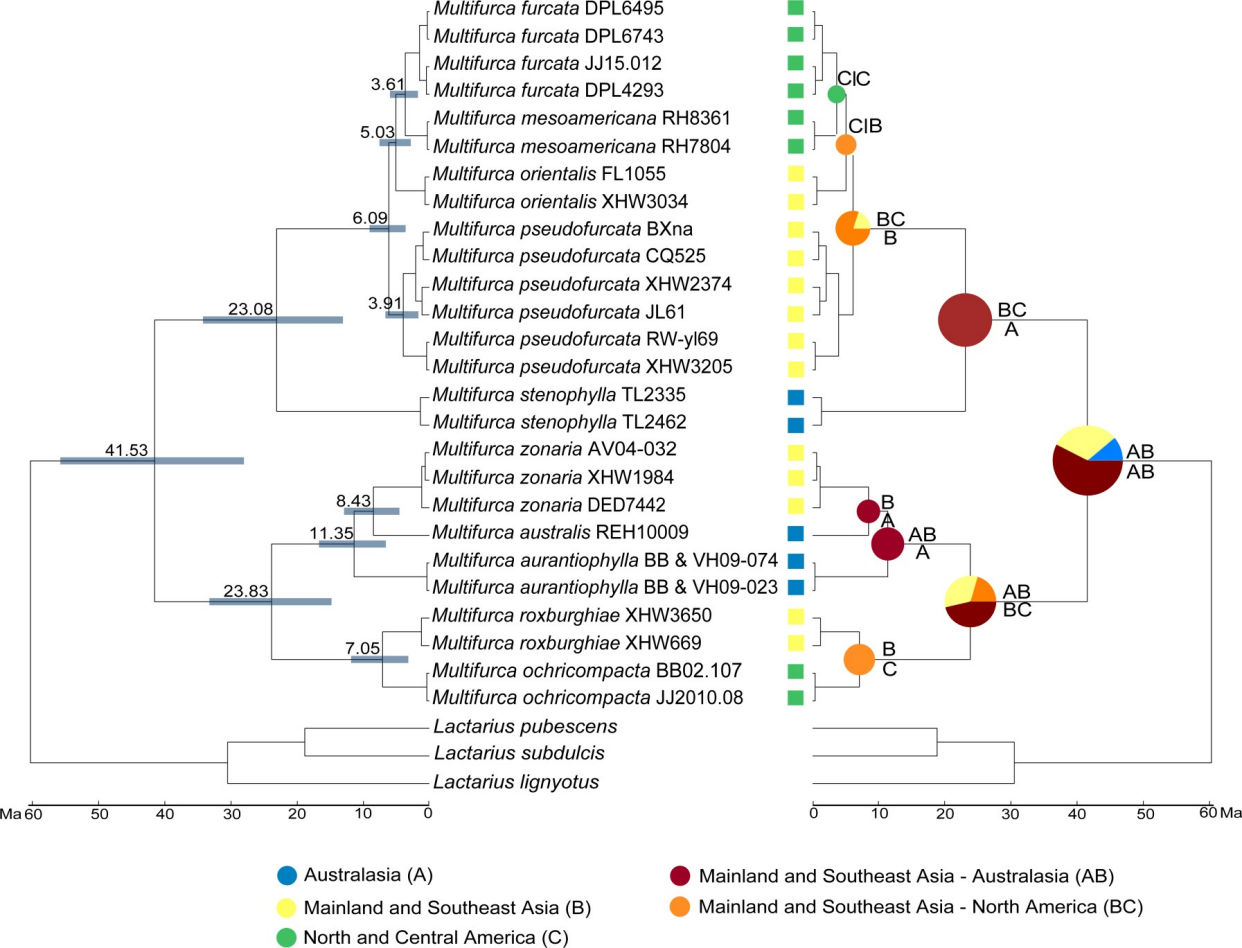
Chronogram, estimated divergence times and ancestral area reconstruction of *Multifurca* generated by the 28S-*rpb2* data. Divergence times were estimated using BEAST and routines by Maximum likelihood-based program LAGRANGE implemented in RASP. Ascomycota-Basidiomycota divergence time of 500–650 Ma was used as calibration point in the molecular clock analysis. Values on the nodes are estimated mean age of the nodes. Greyish-blue bars stand for the 95% highest posterior density. Colored squares by the sample names show the present distribution. Letters above and below the branches represent the inferred best splits by LAGRANGE.

Analyses calibrated with the stem base age of Ascomycota at 500–650 Ma estimated the stem base age of *Multifurca* at 60.32 ± 9.11 Ma (mean ± stdev) and diversification at 41.53 ± 7.22 Ma within the context of the divergence time of Russulaceae at 97.21 ± 13.46 Ma [72.60–124.51 Ma, 95% HPD (95% highest posterior density (HPD)] (S3 Fig). Within *Multifurca*, the two major clades started to diversify appproximately at the same time period: 23.08 ± 5.55 Ma crown age for the lactarioid clade (13.04–34.21 Ma, 95% HPD) and 23.83 ± 4.84 Ma crown age for the russuloid clade (14.78–32.24 Ma, 95% HPD), during the period centered at the early Miocene (Fig 3). Due to unresolved relationships among *M*. *furcata*, *M*. *mesoamericana*, *M*. *orientalis* and *M*. *pseudofurcata*, we did not constrain monophyletic relationships for these four species. BEAST generated a topology grouping American *M*. *furcata* and *M*. *mesoamericana* with East Asian *M*. *orientalis*, with a height of 5.03 Ma. The tMRCA of *M*. *furcata*, *M*. *mesoamericana*, *M*. *orientalis* and *M*. *pseudofurcata* was estimated at 6.09 ± 1.43 Ma (3.56–9.01 Ma, 95% HPD), very close to that of another North America-East Asian terminal clade *M*. *ochricompacta*-*M*. *roxburghiae* (7.05 ± 2.33 Ma, 3.15–11.8 Ma, 95% HPD). The estimated divergence time of the Costa Rican *M*. *mesoamericana* from *M*. *furcata* is at 3.61 ± 1.13 Ma (1.70–5.92 Ma, 95% HPD), partly overlapping with the most recent full closure time of the Isthmus of Panama that is generally accepted as 3.5 Ma [62]. To highlight and focus on the tMRCA of our target group, we only present the *Multifurca* part in Fig 3. The other estimated times are shown in S3 Fig.

### Ancestral area reconstruction (AAR)

The DEC model of LAGRANGE did not give a clear inference of a (single) center of origin for *Multifurca* with significant ML support (Fig 3). Instead, it gave three alternative ancestral areas: Australasia [area A, DEC-probability (DEC-P) 11%], Mainland and southeast Asia (area B, DEC-P 31.4%) and Australiasia—Mainland and southeast Asia (constrained area AB, DEC-P 57.6%). For clade A (the lactarioid clade), joint area AB received full likelihood. For clade B (the russuloid clade), area B (DEC-P 33%), area AB (DEC-P 46.4%) and the joint area Mainland and southeast Asia—North and Central America (area BC, DEC-P 20.5%) were inferred as three alternative ancestral areas. For all clades but the terminal ones, North and Central America were excluded from being ancestral areas.

The formation of the noteworthy Asia—southeastern and Central America disjunct distribution, i.e the species pairs of *M*. *roxhurghiae—M*. *ochricompata* and *M*. *furcata—M*. *mesoamericana—M*. *orientalis—M*. *pseudofurcata*, were inferred to be from two events. For *M*. *roxburghiae—M*. *ochricompacta*, a vicariance event at end of Tertiary (Miocene) was the most likely mechanism (DEC-P 100%), and for *M*. *furcata—M*. *mesoamericana—M*. *orientalis—M*. *pseudofurcata*, the formation was likely due to a dispersal event from the joint area of Asia-America to Asia (for *M*. *pseudofurcata*, DEC-P 80.1%) followed by a vicariance between Asia and America, also at the end of Miocene. The current distributions of the three Australasian species (*M*. *aurantiophylla*, *M*. *australis* and *M*. *stenophylla*) were inferred to be from three different routines. For *M*. *stenophylla*, a dispersal from the joint area AB to a broader area ABC (i.e. to America) and a subsequent vicariance segregating ABC into A and BC might be its mechanism (DEC-P 80.1%). A vicariance event (DEC-P 100%) divided a population at the joint area AB into *M*. *zonaria* (at the area A) and *M*. *australis* (at the area B) and a disperal event (DEC-P 100%) drove the formation of the geographical distribution of *M*. *aurantiophylla* from the joint area AB.

## Discussion and conclusion

### Synapomorphy, phylogeny and worldwide species diversity of *Multifurca*

Including worldwide samples in our morphological and phylogenetic analyses, we confirmed the synapomorphy of *Multifurca* to be a unique combination of three features as concluded by Buyck et al. [3]:

- a pileus context reproducing a much more distinct zonation than the one eventually observed on the pileus surface. Such a zonate context is very rarely known from other Russulaceae.
- regularly forking gills producing an orange spore print. Such regularly forking and yellow gills have also been reported from certain tropical *Lactifluus* [e.g. the Malagasy *L*. *phlebophyllus* (R. Heim) Buyck], while in *Russula* regularly forking gills are also found in some species of *R*. subg. *Malodora* Buyck & V. Hofst. However, none of these species produce an orange spore print.
- remarkably small spores with an inamyloid suprahilar spot. These spores are distinctly smaller than those formed by nearly all other agaricoid Russulaceae, yet comparable in size or even slightly larger than those formed by species of *Russula* subg. *Archaea* Buyck & V. Hofst., which is still assumed to represent the oldest lineage in that genus [11].

In our three-locus phylogeny of *Multifurca*, the genus is composed of two fully supported major clades, in line with previous topologies [3,16,63]. These two clades separate all lactarioid, i.e. latex-producing, species from those that do not produce latex and could thus be described as russuloid. To accentuate the high similarity of all *Multifurca* species, including features of below-ground structures, we prefer to maintain a single genus and recognize each major clade as a distinct subgenus. Our choice of maintaining a single genus is also supported by the placement of *M*. *zonaria* in subg. *Multifurca*, notwithstanding the fact that its morphology would indicate a position amongst the lactarioid species.

GCPSR delimited ten phylogenetic species and analyses on genetic distances added two species in *Multifurca*. In total, twelve species were recognized with DNA data. In *M*. subg. *Multifurca*, morphological differentiation supported all five DNA-based species. In *M*. subg. *Furcata*, we kept five of the seven DNA-based species and merged the two groups of *M*. *pseudofurcata* into one species. The reasons for this “two-in-one” decision are: 1. Although *M*. *pseudofurcata* group 1 met the criterion of an independent lineage (genealogical non-disconcordance, Table 2), they did not form a monophyletic clade in the ITS-28S-*rpb2* tree (S2 Fig); 2. Two different ITS sequences were obtained for sample *M*. *pseudofurcata* XHW2844, one occupying the most basal position in *M*. *pseudofurcata* clade while the other clustering in group 2 (Fig 2). This suggests that recombination may still possibly occur between group 1 and group 2; 3. We could find neither morphological differences between the two groups nor geographical structure within *M*. *pseudofurcata*. Inversely, we think there is sufficient genetic divergence and geographical segregation between the two lactarioid DNA-based species in America, *M*. *furcata* and *M*. *mesoamericana*, to recognize them as two species considering the monophyletic relationship of the two groups lacked significant support in all ML analyses and was only supported in the BI analysis (S2 Fig). The worldwide number of *Multifurca* species recognized in this study is eleven. Each continent has its endemic species.

### Tropical Asia or Australasia as early divergence center of *Multifurca*

AAR did not infer a single center of divergence for *Multifurca* (Fig 3). This is most likely due to the unavailability of the origin center of its sibling genus *Lactarius* and the balanced distribution of its component major clades. However, North and Central America was excluded from being the divergence center and Australasia and Asia received medium (68.6%) to high (89%) marginal likelihood respectively. Analysis of genetic distance in each area showed that Asia and Australasia harbor comparable genetic diversity, higher than North America. In both major clades of *Multifurca*, there is a gradient of genetic distances across the known localities, i.e., from Australasia across Asia to North America, genetic distances decrease. Morphologically, species from Australasia and Asia show higher morphological diversification than those in North America. For instance, the Asian *M*. *zonaria* is the only species in the genus that has both hymenial pseudocystidia and gloeocystidia [3,14,19]. New Caledonian *M*. *aurantiophylla* is the only species in the genus that has mucronate pileocystidia [3,15]. In the lactarioid clade, the Australian *M*. *stenophylla* is the only one that can be morphologically recognized by the larger spores with a well-developed ornamentation [16]. Moreover, in this clade an ITS sequence of a Malaysian sample (GenBank accession KP071201) shows relatively bigger genetic distance than those between the other Asian species and even Asian and American species. All these facts make us believe that tropical Asia, Australasia or somewhere between the two regions might be the key region in the early divergence of *Multifurca* and its two major clades. The final answer to this question will probably depend on an ancestral area reconstruction of its sister genus *Lactarius* or even on a family wide approach.

### Post Cretaceous migrations, dispersals and recent vicariance as possible mechanisms shaping the distribution of *Multifurca*

**Fig 4:**
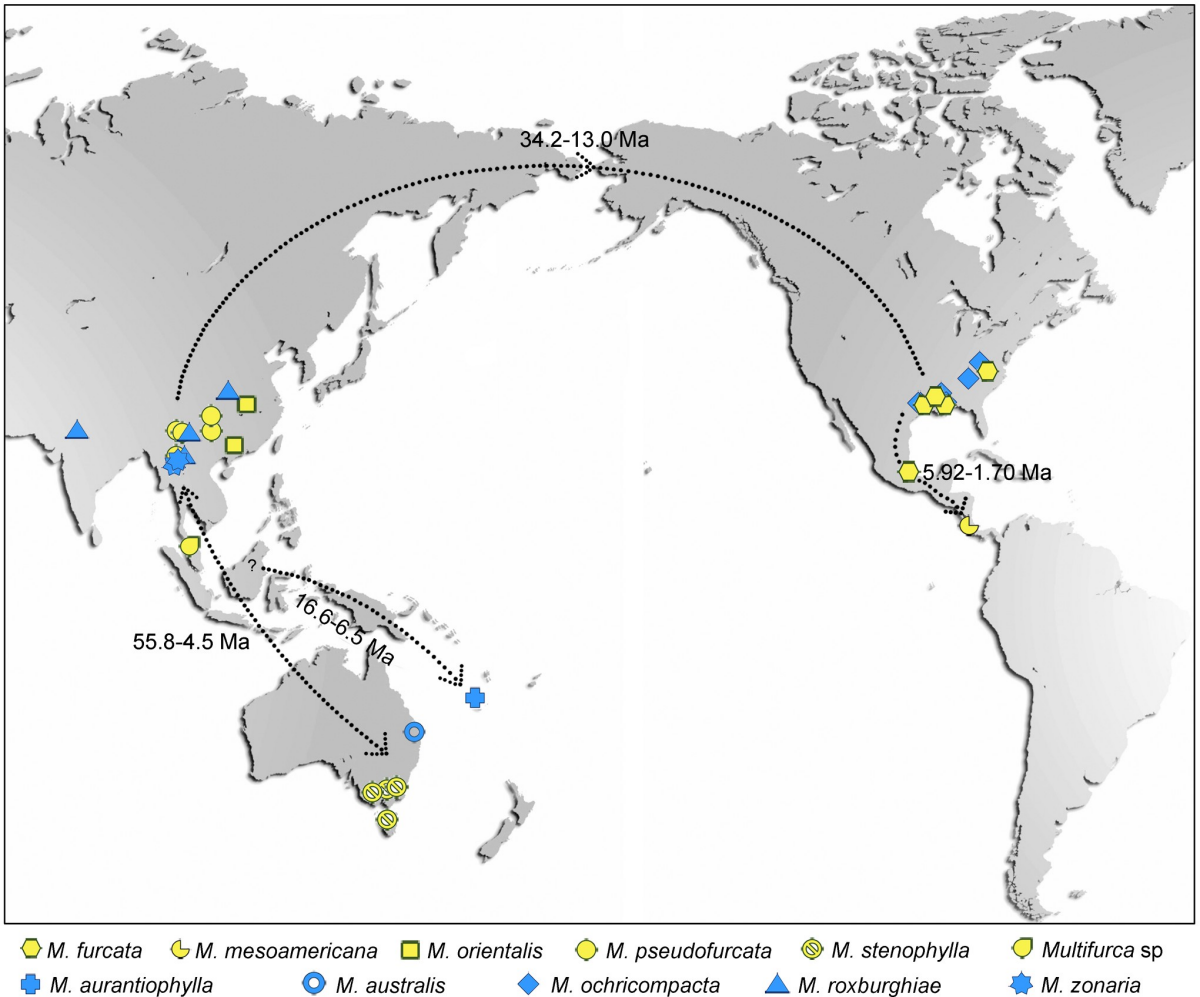
Known worldwide localities of *Multifurca* species and inferred times and routines of dispersals and migrations. Divergence times (95% high posterior density) were estimated using BEAST and routines by Maximum likelihood-based program LAGRANGE implemented in RASP. Question mark means uncertainty of the area of provider. Yellow symbols represent species of *M*. subg. *Furcata* and blue ones of *M*. subg. *Multifurca*. The royalty-free map photo is obtained from www.dreamstime.com (ID 8020640, Jezper).

*Multifurca* is found under Nothofagaceae in New Caledonia and can also occasionally be found with this same host family in Australia [3,16]. However, it was never found in Patagonia, even though these Argentinian Nothofagaceae forests have been explored by quite a number of mycologists. There is also no record of *Multifurca* in Africa or Madagascar. A scenario that *Multifurca* has been extinct in this part of the world seems unlikely given the high local diversity of other ectomycorrhizal genera with a similar tropical distribution such as *Lactifluus* [24] and *Cantharellus* [64]. We can therefore safely conclude that species of *Multifurca* display a typical amphi-Pacific distribution with each continent having its endemic species (Fig 4).

Halling et al. [23] suggested three possible hypotheses that could account for such a distribution: i) long distance dispersal by basidiospores, ii) post-Cretaceous migration of co-symbionts over land bridges with a change or shift in symbiotic partners, and iii) an ancient, Pangaean distribution with little migration since the breakup of Pangaea in the Cretaceous. Previous molecular studies have suggested that these three scenarios are all possible, e.g. *Pisolithus* and some species pairs of Hysterangiales for the first hypothesis, *Solioccasus* and *Hydnum* p.p. for the second, and *Cyttaria* and Tuberaceae for both the first and third) [65–69]. Our analysis using a more ancient calibration point (500–560 Ma for the stem base age of Ascomycota) estimated the origin of *Multifurca* to be centered at the early Paleocene, well after the breakup of Gondwana. Our estimated ages of *Multifurca* and its relatives in Russulaceae and Russulales are slightly older than those given by Looney et al. [63] and Wisitrassameewong et al. [46], although those of the mycenoid and suilloid clades, Agaricales and Boletales are largely in line with the mininum age of the corresponding fossils and estimated ages of these orders using genome data [47,48,70]. Even with these older ages, the third hypothesis, i.e, an ancient, Pangaean distribution with little migration still does not currently fit *Multifurca*.

The decrease in genetic distance from Australasia across Asia to North America argues against a panmictic distribution, and thus against random long distance spore dispersal as the sole mechanism (hypothesis one). Following our AAR results, the corresponding times of divergence and the symbiotic relationships with hosts, we have to accept a complex mechanism that incorporates both medium distance dispersal and post-Cretaceous migration to explain the current distribution pattern.

*Multifurca* comprises two noteworthy biogeographic disjuncts in both subgenera: one between mainland and southeast Asia and Australasia, and the other one between East Asia and eastern North America (Figs 3 and 4). *Multifurca zonaria*, *M*. *aurantiophylla* and *M*. *australis* formed a New Caledonia-Australia-mainland and southeast Asia disjunct pattern, which is an intriguing biogeographic topic [71]. Our AAR inferred (with probability 100%) that the distribution of *M*. *aurantiophylla* in New Caledonia is due to a dispersal event from the joint area of Australasia and mainland and southeast Asia to the current territory (Fig 3). This inference is in line with the consensus that New Caledonia was separated from other land masses early in the upper Cretaceous and has never been connected with them again. Similarly some published data may suggest the same mechanism in ectomycorrhizal fungi between the islands in the South Pacific and other landmasses. For instance, in *Lactifluus*, some samples from American Samoa are found as conspecific with a Papua New Guinean environmental sample [72]. Wang et al. [25] showed that in the *Lactifluus leoninus* lineage, southern China-Thai *L*. aff. *leoninus* and a Papua New Guinean environmental sample shared 97% similarity in ITS sequences. In the genus *Hydnum*, it was found that one sample from Papua New Guinea is nearly identical to some Chinese samples [70]. More recently Han et al. [73] showed at least three independent dispersal events for ancestors of *Strobilomyces* from Southeast Asia to Australasia. Considering the relatively low host specificity of *Multifurca* species or species pairs [3,16,19] we postulate that the dispersal *of M*. *aurantiophylla* might be achieved by island-hopping with the aid of host shifts (Fig 4). Similarly the disjunct distribution between the Australian *M*. *stenophylla* and the rest species of *M*. subg. *Furcata* might be also by means of island-hopping. The northward movement of the Australian landmass during the Miocene is believed to have facilitated these dispersals.

Another interesting biogeographical topic involves the disjunct distribution pattern of the genus between East Asia and eastern North America [20,21,74]. In *Multifurca*, there are two species pairs/complexes that have such a distribution pattern. For the russuloid species pair *M*. *roxburghiae-M*. *ochricompacta*, AAR inferred the disjunct distribution to be due to vicariance from an ancestral population widely distributed in Asia and America. For the lactarioid species complex *M*. *furcata-M*. *mesoamericana-M*. *pseudofurcata-M*. *orientalis*, AAR suggested a first dispersal event from a widely distributed population in Asia-North America to Asia (for *M*. *pseudofurcata*) and then a vicariance from an ancestral population in Asia and America. This inference must be strongly influenced by the sibling relationship between American *M*. *furcata* and *M*. *mesoamericana* and Asian *M*. *orientalis*. Actually this topology was only produced by BEAST analysis and never received support in any other phylogenetic analysis. Therefore we would suggest this topology is an artifact and the relationship among these four species is actually equivocal. Taking account of such relationship and the shared INDELs and degenerated base pairs among these species (Fig 1), we would also like to suggest a scenario that a first vicariance between Asia and North America and a subsequent but very close migrations between eastern and western parts of Asia and southeastern North America and Central America respectively. In both species pairs/complexes, the widely distributed ancestral Asia-North American populations seem to have formed at the time between Miocene to Pliocene. The relatively warm climate in Miocene [75] and the presence of Beringia at that time [21] are believed to keep the Tertiary populations possible. The time of vicariance between Asia and North America was estimated to start around at the end of Tertiary, when the warm and humid climate was cooling down. For *Multifurca*, a basically thermophilic group, the decrease of temperature would make the widely distributed population of the ancestor of *M*. *furcata—M*. *orientalis—M*. *pseudofurcata—M*. *orientalis* segregated, by pushing each population to the south. For *M*. *furcata*, a subsequent migration from North America via Mexico to Central America and speciation of *M*. *mesoamericana* is obvious. Our dating estimated the divergence between the North American *M*. *furcata* and Costa Rican *M*. *mesoamericana* at the late Pliocene to the Quaternary (3.61 ± 1.13 Ma, 1.70–5.92 Ma, HPD 95%), perfectly in line with the significantly increased biotic exchange between North and South America around 3 Ma [62]. The vicariance and migration must have taken place very fast, so that we see quite many shared polymorphisms in the ITS data (Fig 1), the weak conflicts between the ITS and 28S genealogies (S2 Fig) and the numerous degenerated sites and poor differentiation in the *rpb2* data. This could also explain why in the American-Asian *M*. *furcata* complex there is no absolute split between Asia and Eastern North America, quite different from the findings in plant examples [76–78]. In Russulaceae there are at least two more groups that show such distribution pattern: *Lactifluus* sect. *Gerardii* (A.H. Sm. & Hesler) Stubbe and *Russula compacta* species complex [7,79]. Moreover in *L*. sect. *Gerardii*, similar equivocal relationship was found in some terminal clades [7]. The biogeographical analyses of the species pairs of *Multifurca* here will be of special reference for these biogeographically interesting groups.

### Ectomycorrhizal associations of *Multifurca* species

The presumed ectomycorrhizal nature of the species composing *Multifurca* is still circumstantial and, notwithstanding several efforts by one of us (BB), dual symbiotic root structures have not yet been demonstrated, but the morphology of rhizomorphs emanating from the stipe base confirm the typical features of the family [11]. All the *Multifurca* specimens studied in this work were collected near putative ectomycorrhizal trees: in the northern hemisphere mainly Pinaceae and Fagaceae and to a lesser extent Dipterocarpaceae, while Nothofagaceae and Myrtaceae were likely tree hosts in the southern hemisphere. The Chinese specimens were all found under mixed forests with trees of *Pinus* subg. *Pinus* and Fagaceae or in pure pine forest, such as *M*. *roxburghiae*, which was originally described from *Pinus roxburghii* forests in Himalayan India [3] and re-collected in this same habitat at the moment of submission of this paper (K. Das pers. comm.). *Multifurca zonaria* was associated with fagaceous trees (*Castanopsis* spp.) in China [19], but with dipterocarp hosts in Thailand [14]. The American *M*. *furcata* and *M*. *ochricompacta* were all collected in fagaceous forests dominated by *Quercus* spp., although for both North American taxa, beech and in some cases also pine were close by [18] (Buyck pers. obs.). Nothofagaceae were the likely hosts for the New Caledonian *M*. *aurantiophylla* and for some collections of Australian *M*. *stenophylla*, although Myrtaceae (including *Eucalyptus* spp.) were clearly associated with some other collections of the latter, and apparently the only possible host for the single specimen of *M*. *australis* described here. It is most parsimonious to infer that *Multifurca* is a putative ectomycorrhizal group, using these two evidences: 1). Both our phylogenetic analysis and previous phylogeny of Russulaceae [3,7,9,24] show that *Multifurca* is nested in the core clade of Russulaceae that exclusively includes ectomycorrhizal species; 2). All the tree families/genus mentioned above have ages older than *Multifurca* [71,80,81].

Although based on the available data we are not able to infer the ancestral host of the genus and its major clades, the remote relationships of the putative hosts of the species pairs and even the same species (i.e. relatively low host specificity) strongly suggest host shifts during the diversification of the genus, which have been reported in many ectomycorrhizal groups [64,82,83]. If the symbiotic relationships of *Multifurca* species and these trees are eventually clearly demonstrated, the medium distance dispersals and migrations among America, Asia and Australasia and the present amphi-pacific distribution pattern of *Multifurca* will be better understood.

### Taxonomy

**Fig 5:**
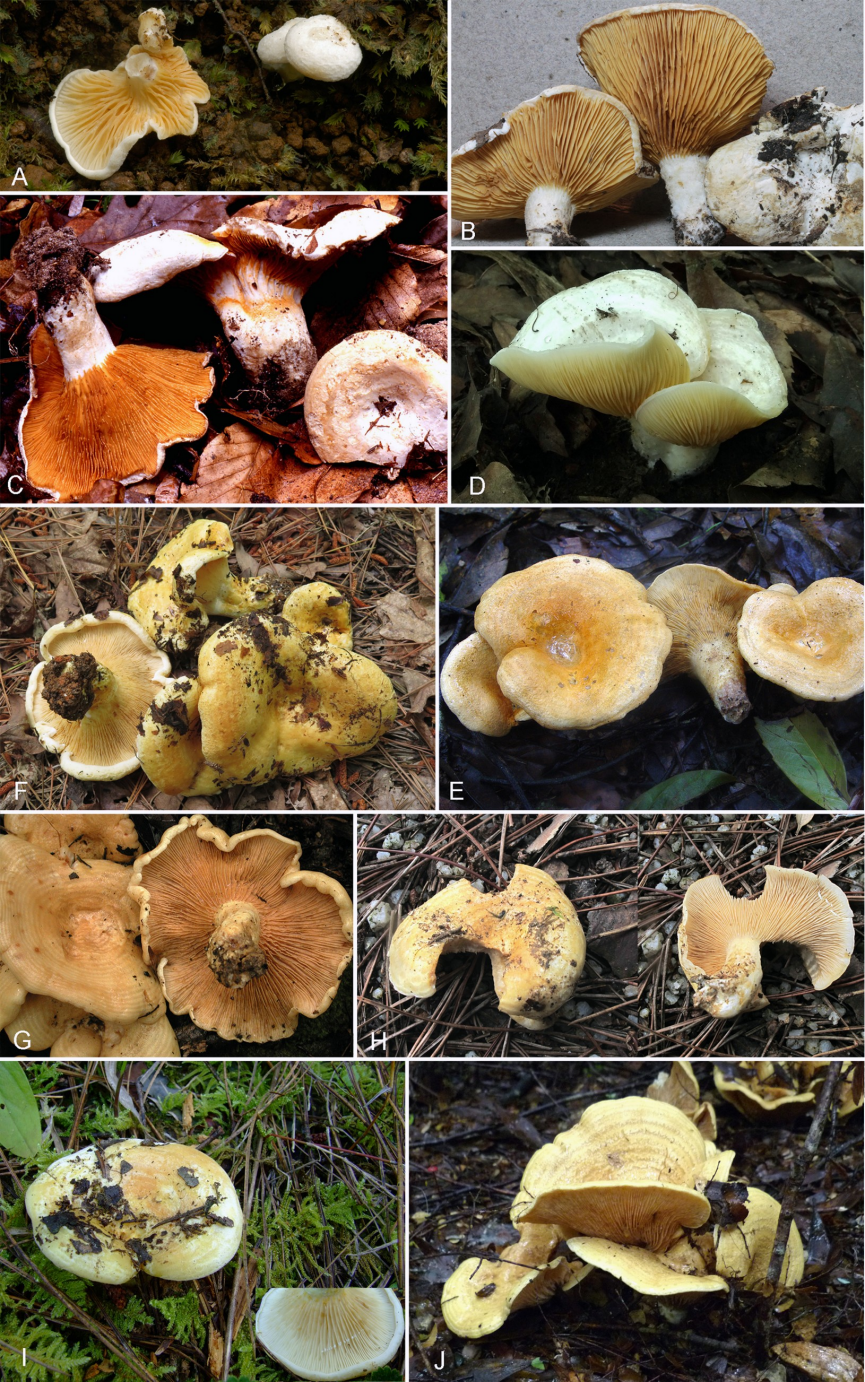
Basidiocarps of species of *Multifurca*. A-E. *Multifurca* subg. *Multifurca*: A. *M*. *aurantiophylla* (BB09.073); B. *M*. *australis* (REH10009); C. *M*. *ochricompacta* (BB02.118); D. *M*. *roxburghiae* (XHW3650); E. *M*. *zonaria* (XHW1984); F-J *Multifurca* subg. *Furcata*: F. *M*. *furcata* (JJ15.012); G. *M*. *mesoamericana* (REH8361); H. *M*. *orientalis* (XHW3034); I. *M*. *pseudofurcata* (XHW3205); J. *M*. *stenophylla* (TL2335).

**1. *Multifurca* subgenus *Multifurca***, Fig 5A–5E

Although the species of this subgenus can be considered ‘russuloid’, as they do not exude latex upon injury, they do share several features that make them more similar to *Lactarius*, rather than to *Russula*. Indeed, the often zonate cap and scrobiculate stipe are characteristic of *Lactarius*, and so is the lamellar trama that is mainly composed of hyphae whereas sphaerocytes are dominant in the lamellar trama of *Russula* and most *Lactifluus*. *Multifurca zonaria* even has unequivocal hymenial pseudocystidia. *Multifurca aurantiophylla* is the only species of the genus that has well-differentiated pileocystidia, similar to many species of *Russula*.

The hymenial macrocystidia in *Multifurca* subg. *Multifurca* (except *M*. *zonaria*) are of two types. The first type concerns very voluminous cystidia that are deeply imbedded in the lamellar trama where these may sometimes inflate remarkably and emerge at the surface through a long neck. This type is unique among agaricoid Russulaceae as they are identical to those of some corticioid *Gloeocystidiellum* species. The second type is ‘normal’ minutely mucronate hymenial gloeocystidia that are so typical for most *Russula*. This second type can sometimes be extremely rare and is therefore easily overlooked, e.g. in some specimens of *M*. *ochricompacta* and *M*. *roxburghiae*. It is interesting to note that the minutely mucronate gloeocystidium type is absent from other parts of the fruiting body, but becomes abundant in the below-ground structures of all species studied in this respect: *M*. *aurantiophylla* [11], *M*. *australis* (this paper), *M*. *zonaria* (this paper) and even in the lactarioid *M*. *furcata* (this paper).

Type species:

**1.1 *Multifurca ochricompacta*** (Bills & O.K. Miller) Buyck & V. Hofst., Fungal Diversity 28: 37. 2008. Fig 5C

*Basionym*: *Russula ochricompacta* Bills & O.K. Mill., Mycologia 76: 976. 1984.

*Examined material*: UNITED STATES OF AMERICA. North Carolina: Buncombe Co., near Asheville, 16 July 2004, B. Buyck 04.278 (PC). Texas: Newton Co., Bleakwood, 262 CR 3062, state highway 87, 5 September 1996, D.P. Lewis’ property, in Oak-Gum floodplain, D.P. Lewis 5697; ibid., 12 July 1997, D.P. Lewis 5824; ibid., 30 July 2002, D.P. Lewis 6609; ibid., 5 July 2003, D.P. Lewis 6738 (all PC, F); near D.P. Lewis’ property, in Pine uplands, 25 September 2009, D.P. Lewis 9318 (PC, F); ibid., 4 July 2002, B. Buyck 02.107; Canyon rim trail, near Mayflower along state highway 87, 5 July 2002, B. Buyck 02.118; ibid., 21 June 2014, B. Buyck & V. Hofstetter 14.001, 14.013, 14.014, 14.015 (all PC); ibid., bottomland hardwoods with water oak and laurel oak, B. Buyck 07.010 (PC); ibid., closer to beech, 24 July 2007, B. Buyck 07.060 (PC); Hardin Co., Big Thicket Nat Pres., Teel Rd, in mixed pine-hardwood forest, 3 October 2009, D.P Lewis 9395 (PC, F). Mississippi: Stone Co., near Wiggins, Black Creek, parking lot tuksakane trail, Esoto natl forest, 11 July 2014, B. Buyck & V. Hofstetter 14.177 (PC). Arkansas: Perry Co., Lake Sylvia Area, camping, 14 June 2010, J. Justice 2010.08 (PC)

*Commentary*: This American species might be the most (locally) common species in the genus. One of us (BB) has found it frequently in bottomland hardwoods and Beech-Magnolia-Loblolly Pine slope forests. It differs from all other species in this subgenus by its lower spore ornamentation.

Additional species:

**1.2 *Multifurca aurantiophylla*** (Buyck & Ducousso) Buyck & V. Hofst., Fungal Diversity 28: 37. 2008. Fig 5A

*Basionym*: *Russula aurantiophylla* Buyck & Ducousso, Cryptogamie Mycologie 25:127. 2004.

*Examined Material*: NEW CALEDONIA. Northern Prov.: Koniambo, elev. 720–850 m, on ultramafic soil, under *Nothofagus balansai* and *N*. *codonandia*, 17–19 March 2009, B. Buyck 09.023 (PC0723661), 09.035 (PC0125024), 09.073 (PC0125022), 09.074 (PC0723662), 09.110 (PC0125021), 09.116 (PC0125023), 09.119 (PC0142421); ibid., Koniambo, near entry Trazy forest, under *N*. *codonandra*, 9 April 2009, B. Buyck 09.345 (PC0723657)

*Commentary*: This species is by far the smallest species of all *Multifurca* (Fig 5A), in our opinion a consequence of the toxic metallic soils in which the host trees are living, because the exceptional small size is an almost general phenomenon for most of the local ectomycorrhizal fungi [84]. This species often has a strongly eccentric to laterally implanted stipe, which again, might be related to the often steep to vertical slopes on which this species often sporulates. This is the only species in the genus that has well-developed pileocystidia.

**Fig 6:**
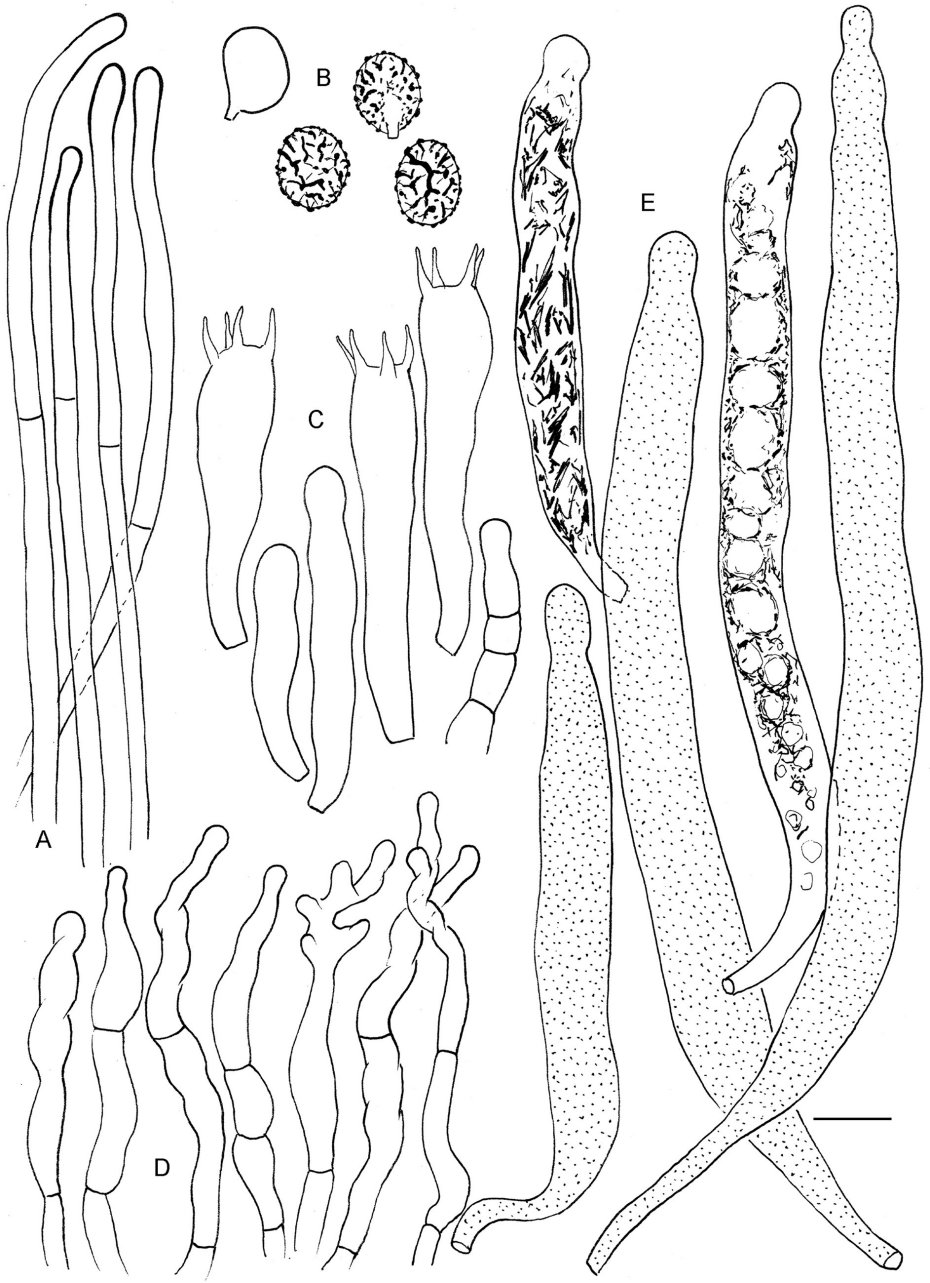
Microscopical drawings of the basidiocarps of *Multifurca australis*. A. Hyphal terminations of the pileipellis. B. Spores; C. Basidia and basidiola; D. Marginal cells; E. Hymenial gloeocystidia. Scale bar = 10 μm for all except for 5 μm for spores.

**Fig 7:**
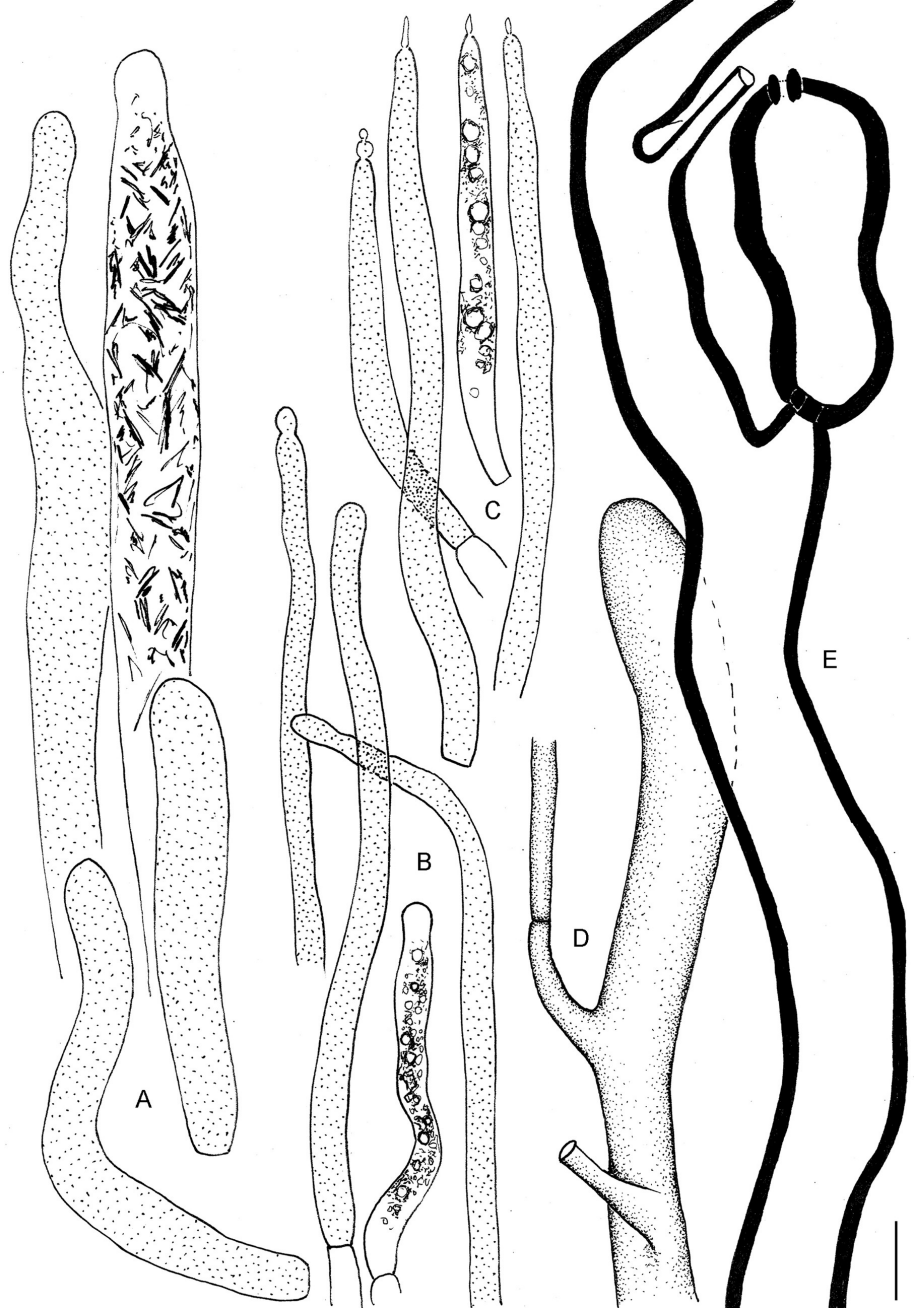
Microscopical drawings of the rhizomorphs of *Multifurca australis*. A. Gloeocystidia of the stipe surface; B. Gloeocystidia in the trichoid bundles of hyphal extremities that occupy most of the lower stipe. C. Myceliocystidia; D-E. Vessel-like hyphae (sensu Agerer) of the stipe base-soil transition, in ‘E’ showing wall thickness in section. Scale bar = 10 μm.

**1.3 *Multifurca australis* Halling, T. Lebel & Buyck,** sp nov. Figs 5B, 6 and 7.

MycoBank number: MB 825354

*Holotype*: AUSTRALIA. Queensland: near Landsborough, north of Brisbane, gregarious on soil in dry sclerophyllous vegetation with *Eucalyptus* sp., *Melaleuca* sp., *Allocasuarina* sp. and various Myrtaceae, 18 February 2015, R.E. Halling 10009 (BRI AQ0799405; isotypes at ISO, NY, PC, MEL).

*Diagnosis*: Differs from *M*. *aurantiophylla* in the field because of the larger basidioma size, the more central stipe and its association with Myrtaceae; differs from all other whitish species of the genus because of the one-type gleoecystida that are never ventricose.

Pileus 4–8 cm broad, depressed to deeply depressed on disc, dry white then pale ochre, finely subtomentose to finely matte, azonate. Flesh white, unchanging, with curved, light and dark zonations in some, odor mild, taste acrid (within 10–15 sec). Latex apparently absent, but scant watery exudate evident in one basidiome. Lamellae adnate to subdecurrent, crowded, forked in 3–4 ranks, light orange (5A–B6,5) at first, then yellow ochre (5C7) with age, with even edges. Stipe 3–4 cm long, 1–2 cm broad, equal, central to slightly eccentric, dry, white, sublanose to tomentose, rarely subscrobiculate, with interior white to a very pale ochraceous, otherwise unchanging, becoming hollow. Spore print faint, but definitely orange-tinted.

Spores shortly ellipsoid, small, (5.0) 5.1–***5*.*36***–5.6 (5.8) × (3.7) 3.9–***4*.*17***–4.4 (4.6) μm, Q = (1.14) 1.21–***1*.*29***–1.37 (1.44); ornamentation subreticulate, low (ca 0.2 μm high), predominantly composed of ramifying crests, but often also with occasional laterally prolonged, convex warts and subtle line connections, the whole forming a subreticulate to almost reticulate pattern of variable density; some clearly ill-developed spores lacking the subreticulate ornamentation but beset with widely dispersed and strongly amyloid, blunt, isolated warts or even droplet-like pustules; suprahilar spot relatively small, inamyloid. Basidia (35) 40–50 × 7–9 μm, clavulate, four-spored, with rather slender, long sterigmata, 5–6 × 1 μm; basidiola slender, subcylindrical to clavulate; intermixed with many very slender, irregular, sometimes terminally branching cells that are clearly not basidiola but clearly visible at the hymenial surface. Hymenial gloeocystidia very abundant on sides and edges of the gills, varying from hardly emergent to projecting up to 20–30 μm beyond the basidia, robust and of very variable size, originating sometimes from deep within the trama, (40) 80–150 (250) × (6) 8–12 μm, fusiform, subcylindrical, often slightly constricted subapically and subcapitate to rarely appendiculate, with a usually short neck, shortly pedicellate, filled with coarsely refringent-amorphous contents organized around circular cavities, that become then slowly coarsely crystalline, partially blackening in SV. Marginal cells slender, in length similar to basidiola, but not wider than 6 μm, somewhat undulate or repeatedly constricted in outline, usually tapering toward the tip, sometimes diverticulate. Lamellar trama a dense tissue of slender and narrow, strongly branching, filamentous hyphae of similar diameter (ca 2–3 μm), intermixed with the protruding bases of the gloeocystidia and some dispersed oleiferous fragments that are strongly and irregularly inflated-nodulose or variously constricted, also with some rare cystidioid hyphae. Pileipellis one-layered, poorly differentiated, hardly gelatinized, entirely orthochromatic in cresyl blue, near the very pileus surface forming a relatively thick tissue of very densely interwoven and very narrow, hardly 3 μm wide, and regularly cylindrical hyphae with simple, obtuse-rounded extremities; there also thin-walled or with slightly refringent walls near the very tips, but towards the lower pileipellis and underlying context often distinctly coated with refringent, mucus-like substance; inside the hyphae often with sparse refringent granules or inclusions. Well-differentiated pileocystidia and lactifers not observed. Stipitipellis equally undifferentiated, but in its lower portion developing large trichoids composed of very long and slender, cylindrical hyphae of identical diameter, some long caulocystidia of ca. 4 μm diam. present in these trichoid fascicules, but particularly abundant below the surface in the lower stipe and there often very large (similar in diam. to hymenial cystidia) and with first granular-amorphous, refringent, then coarsely crystalline contents; thick-walled vessel-like hyphae of variable diam. and length present inside lower stipe, also continuing in onset of rhizomorphal tissue below, some empty, others filled with homogeneous dense substance. Oleiferous hyphae abundant, similar as in pileus trama. Ladder-like hyphae not observed. Clamp connections absent. Below the stipe developing mycelial tissue with long and slender, aculeate and minutely mucronate gloeocystidia, 4–5 μm wide.

*Commentary*: The hymenial gloeocystidia of *M*. *australis* are never ventricose, i.e. they do not inflate in the part that is embedded in the subhymenium or lamellar trama, while they inflate moderately in *M*. *aurantiophylla*, and strongly so in *M*. *ochricompacta* and *M*. *roxburghiae*. The Australian species differs further from *M*. *aurantiophylla* in the field because of the (much) larger basidioma size and more central stipe (Fig 5B), as well as in its association with Myrtaceae; microscopically also in the smaller size and lower ornamentation of spores, the more regular and more slender hyphal endings in the pileipellis and absence of minutely mucronate, small hymenial gloeocystidia. Although *M*. *australis* does not possess minutely mucronate gloeocystidia in its fruiting body, it produces these on the below-ground parts (mycelium, rhizomorphs). They are nearly identical in form to those of *M*. *aurantiophylla* but lack the secondary septation as observed in the latter (Fig 7C, compare with ref. 11). The gloeocystidia in the lower stipe of *M*. *australis* do possess distinct contents (Fig 7A and 7B) and are therefore clearly differentiated contrary to the pileus where these are not apparent.

**1.4 *Multifurca roxburghiae*** Buyck & V. Hofst., Fungal Diversity 28: 38. 2008. Fig 5D *= Russula grossa* sensu Bills & Pegler 1988 ac sensu Saini & Arti 1982, non Berkeley 1851.

*Specimens examined*: CHINA. Henan Prov.: Luanchuan Co., Laojun Mt., Tenglongyu scenic section, elev. 1250–1360 m, N30°44'08.17'' E111°39'13.70'', under mixed forest of *Pinus tabuliformis* and *Quercus* sp., 13 August 2015, X.H. Wang 3650 (HKAS 89924, KUN). Yunnan Prov.: Tengchong Co., Zhonghe, Tiegongshan, 05 August 1977, X.L. Zeng 576 (HKAS 3416, KUN); Wuding Co., Chadian, under *Pinus yunnanensis*, elev. 2380 m, 6 August 1998, X.H. Wang 669 (HKAS 39328, KUN).

*Commentary*: Buyck et al. [3] gave a detailed description based on the holotype collection, which was originally mistaken for *R*. *grossa*, an unrelated species. The Chinese specimens are highly similar to the Indian material, but differ essentially in the somewhat smaller spores [(5.0) 5.5–*5*.*72*–6.5 × 4.0–*4*.*46*–5.0 μm versus (5.9) 6–*6*.*39*–6.8 (7.1) × (4.3) 4.5–*4*.*79*–5.1(5.5)].

**1.5 *Multifurca zonaria*** (Buyck & Desjardin) Buyck & V. Hofst, Fungal Diversity 28: 37. 2008. Fig 5E

*Basionym*: *Russula zonaria* Buyck & Desjardin, Cryptogamie Mycologie 24: 112. 2003.

*Specimens examined*: CHINA. Yunnan Prov.: Jinghong Pref., Dadugang, under *Castanopsis* forest, 30 August 2004, X.H. Wang 1790 (HKAS 47712, KUN); Jinghong, Jinuoshan, elev. 1000 m, under *Castanopsis* forest, 9 July 2006, X.H. Wang 1984 (HKAS 51464). THAILAND. Chiang Mai Prov.: Doi Suthep National Park, Sangasabhasri Lane to Huai Kok Ma Village, elev. 1200 m, 3 July 2002, D.E. Desjardin 7442 (SFSU, BBH, PC, holotype); ibid., 7 June 2006, R.E. Halling 8797 (NY817442).

*Commentary*: We refer to Buyck & Desjardin [14] and Wang & Liu [19] for a detailed description and illustrations of this species. These Chinese specimens differ from the type in the stronger, more crested, less catenated spore ornamentation. Rare hymenial gloeocystidia were observed in the Chinese specimens, while these were reported as absent or doubtful for the type collection. Examination of another collection from the type locality (REH8797) allowed us to study rhizomorphs emanating from the stipe base and mycelial tissue. The rhizomorph surface of the stipe base is very similar to the one illustrated for *M*. *aurantiophylla* [11], i.e. covered with dispersed, slender, aculeate and minutely mucronate gloeocystidia. In *M*. *zonaria*, however, these gloeocystidia lack secondary septa, similarly as in *M*. *australis*. This type of gloeocystidia is the only one present in the trichoid fascicules at the finer endings of rhizomorphs. Besides these aculeate and very slender gloeocystidia, the surface of the broader rhizomorph parts also carries dense clusters of much shorter, but wider and mostly distinctly capitate gloeocystidia, very similar in form to the typical hymenial cystidia in Russulaceae. Inside the rhizomorphs vessel-like hyphae, short ladder-like hyphae and lactifer-like hyphae could be observed.

This species differs considerably from all other species in *M*. subg. *Multifurca* because of its darker pileus color and the almost vein-like, low gills-folds, giving it the typical habit of species in the other subgenus (see Fig 5E), further accentuated by the conspicuous presence of pseudocystidia. Although this species morphologically bridges both subgenera, our phylogeny places it firmly in subg. *Multifurca* where it does not even occupy an isolated position as one might expect from its different morphology.

**2. *Multifurca* subgenus *Furcata* Buyck & X. H. Wang subg. nov.**, Fig 6F–6J

*MycoBank number*: MB 825355

*Type species*: ***Multifurca furcata*** (Coker) Buyck & V. Hofst., Fungal Divers. 28: 37. 2008

*Basionym*: *Lactarius furcatus* Coker, J. Elisha Mitchell Sci. Soc. 34: 18. 1918.

*Diagnosis*: Differs from *M*. subg. *Multifurca* in the latex exudation on injury, yellowish basidiocarps, absence of gloeocystidia, presence of hymenial pseudocystidia and the mostly less reticulate, lower spore ornamentation.

*Commentary*: All the species of this subgenus have abundant lactifers ending in pseudocystidia and the pileipellis is an ixocutis [16–19,85]. Except for *M*. *stenophylla* that has bigger spores, all of the species in the subgenus are more or less cryptic.

**Fig 8:**
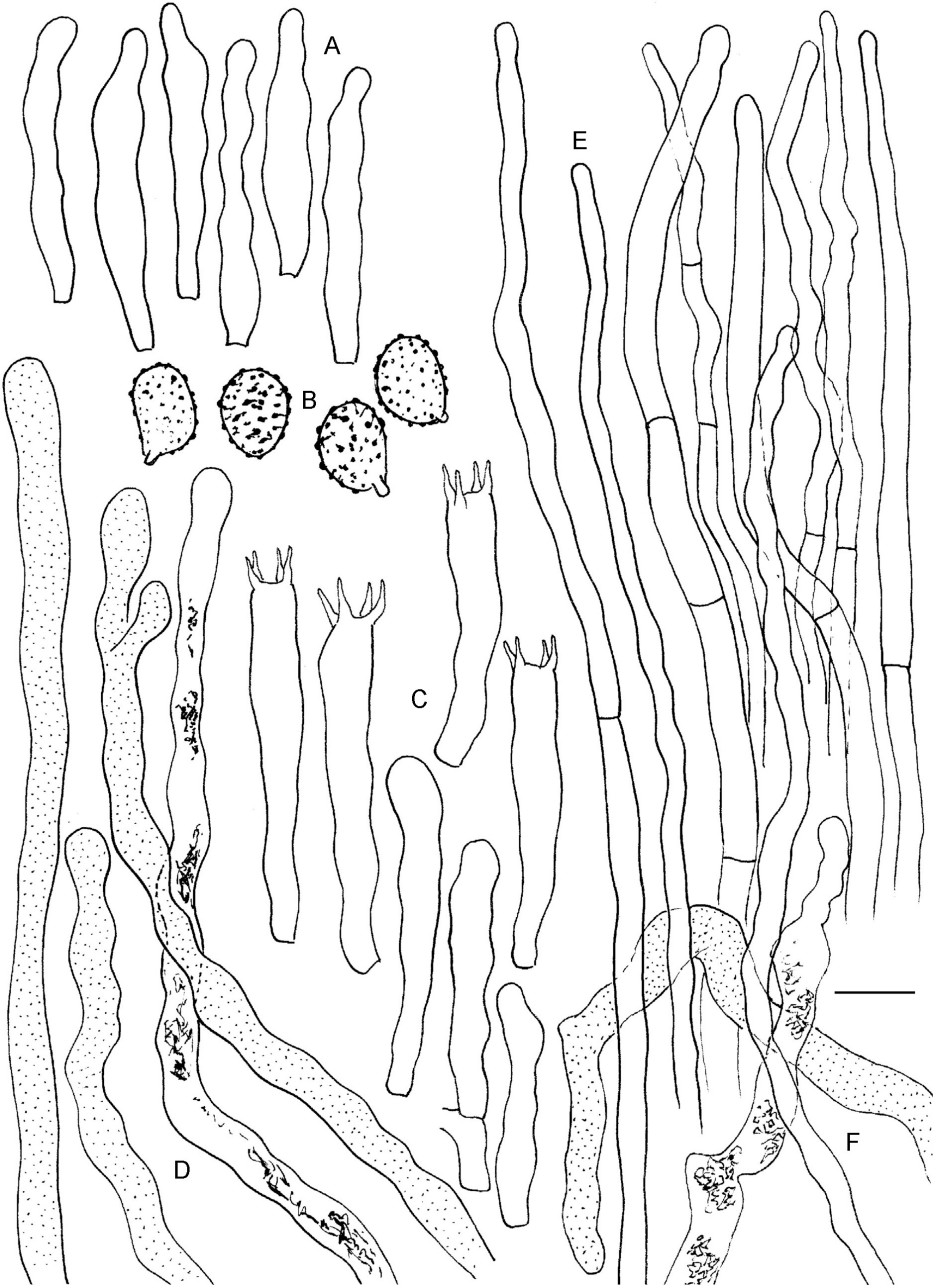
Microscopical drawings of the basidiocarps of *Multifurca furcata*. A. Marginal cells; B. Spores; C. Basidia and basidiola; D. Pseudocystidia of the hymenium; E. Hyphal terminations of the pileipellis; F. Pseudocystidia on cap surface. Scale bar = 10 μm for all except for 5 μm for spores.

**2.1 *Multifurca furcata*** (Coker) Buyck & V. Hofst., Fungal Divers. 28: 37. 2008. Figs 5F and 8

**Lectotypification**: Plate 14 [*Lactarius furcatus* No 2232. TYPE], in J. Elisha Mitchell Sci. Soc. 34(1). 1918, **lectotypus hic designatus**.

**Epitypification**: UNITED STATES OF AMERICA. Texas, Newton Co., Bleakwood, in stream hardwood bottomlands, D.P. Lewis’ property, 7 August 2003, D.P. Lewis 6743 (F, **Epitypus hic designatus** !).

Cap up to 9 cm broad, when young slightly depressed in the center and already irregular in outline with a lobed or wavy and strongly inrolled margin, becoming infundibuliform at maturity with the margin plane or narrowly revolute, not becoming striate; surface covered by a subtle, white, felted tomentum that can locally take web-like to almost membranous aspects over the disc but is distinctly hirsute-fibrillose at the margin, only moderately viscid when wet, yellowish ochraceous with locally salmon orange tints, with faint concentrical zonation. Gills decurrent, quite crowded, very narrow, < 2 mm high, repeatedly forking, off-white when young, then fleshy-ochraceous with a tinge of salmon. Stem 2–3 cm. long and about 1.3 cm. thick, firm, solid and tough but the central part often narrowly and irregularly hollowing out over most of its length, surface covered by the same white tomentum as the cap but densely pitted-scrobiculate with light yellowish to ochraceous spots of variable size, stipe base not rounded, but clearly irregular. Flesh white and firm, in the cap with distinct concentrical watery-gray zonation throughout, up to 7 mm thick near stem; Odor weak. Milk moderately abundant, white, then slowly a distinct glaucous gray and remaining this color indefinitely; moderately acrid. Spore print pale salmon.

Spores ellipsoid, (4.4) 4.9–***5*.*23***–5.5 (5.6) × (3.5) 3.8–***4*.*09***–4.3 (4.6) μm, Q = (1.14) 1.21–***1*.*28***–1.35 (1.39); ornamentation overall low and faint, less than 0.2 μm high, densely punctuated with isolated irregular warts of variable diam., some hardly visible, without distinct linear connections nor ridges, but some warts laterally prolonged and comma-like; suprahilar spot ill-differentiated, verruculose, not amyloid or very rarely graying in its distal part. Basidia 34–45 (48) × 5–6 (7) μm, narrowly clavulate to subcylindrical, 4-spored with slender sterigmata. Hymenial gloeocystidia absent. Pseudocystidia common, (3) 4–5 (7) μm wide, rarely projecting beyond the basidia, cylindrical but often undulating or repeatedly but gently constricted, with obtuse-rounded or slightly tapering apex, with dense, yellowish-granular contents. Marginal cells occupying an almost sterile lamellar edge, more slender and irregular in outline compared to basidiola, often apically narrowing. Lamellar trama composed of hyphae and lactifers, in the more distal part and pileus context with distinct rosettes. Pileipellis an ixocutis, weakly gelatinized, 250–350 μm thick at mid-radius, of loosely interwoven, very thin-walled and narrow hyphae, well delimited from the underlying trama; hyphal terminations very narrow, ca. 2 μm wide, intensely yellowish and refringent, very long (sometimes up to several 100 μm), slender, not aggregated in trichoid bundles, mostly somewhat wavy-tortuous in outline, with thin, hardly visible septa, more densely septate near the basal cell, rarely also higher up. Lactifers abundant, especially in the lower cutis and underlying trama, yellowish and refringent, with some granular to crystalline contents, some ending near the pileipellis surface as ill-defined pseudocystidia that can hardly be differentiated from the other hyphal extremities, sometimes somewhat constricted apically, 2–3 μm diam. Stipitipellis composed as the pileipellis, toward the base with hyphal terminations often aggregated in bundles, with very abundant lactifers, all of very similar diam., mostly 4–6 μm diam., obtuse rounded at the apex, or also frequently subapically constricted and mucronate, forming 1 or 2 successive knobs; at the soil transition even more minutely mucronate and there very similar to the cystidial apices of myceliocystidia for species in subg. *Multifurca*, lactifers and pseudocystidia abundant also below the stipe tissue. Clamp connections absent everywhere.

*Additional material examined*: UNITED STATES OF AMERICA. Arkansas: Perry Co., near the Lake Sylvia Spur, Ouachita National Recreation Trail, at the crossing with Highway 324, across the highway from the paved parking lot adjacent to the Nature trail, GIS 34 52 3.748, -92 49 20.507, 22 May, 2015, J. Justice 15.012 (PC 0723659). Texas: Hardin Co., Big Thicket National Preserve, Kirby trail, under *Pinus*, 17 June 1984, D.P. Lewis 3689 (TENN); Newton Co., Bleakwood, in stream hardwood bottomlands, D.P. Lewis’ property, D.P. Lewis 6330 (F, PC); ibid., 6 June 2001, D.P. Lewis 6456 (LSUM); ibid., 7 Aug. 2003, D.P. Lewis 6743 (F). Mississippi: Desoto Nat. Forest, Black Creek trail, in mixed woods, 16 July 1989, leg. J. Perry, D.P. Lewis 4293 (F); ibid.,14 July 2001, D.P. Lewis 6495 (F).

*Commentary*: Although *M*. *furcata* shares the same habitat and area of distribution with *M*. *ochricompacta*, it is much rarer than the latter species. Buyck et al. [3] discussed *M*. *furcata* on the basis of sequence data obtained for *M*. *mesoamericana*, which is described as a new species below. Given the prevalence of cryptic species in the *M*. *furcata* species complex and even several ITS types of *M*. *furcata* in North America, to fix the precise application of the name *M*. *furcata*, epitypification based on DNA data is needed.

When studying the mycelium-stipe transition, we found long and slender pseudocystidia with apices reminiscent of the typical aculeate-mucronate gloeocystidia in below-ground parts of species of subg. *Multifurca*. However, we were unable to find well-developed rhizomorphs. So far, there are no other observations made for below-ground structures of species in subg. *Furcata*.

**2.2 *Multifurca mesoamericana* Buyck & Halling, sp. nov.** Fig 5G

*MycoBank number*: MB 825353

*Holotype*: COSTA RICA. Puntarenas, Coto Brus, Las Mellizas, La Amistad Lodge, near Parque Internacional La Amistad, 3. July 1998, R.E. Halling 7804 (NY792793).

*Diagnosis*: Differs from *M*. *furcata*, in the somewhat less fleshy habit, the distinctly zonate, more strongly viscose and less brightly colored cap, and its association with endemic *Quercus* in Central America; it further differs molecularly in ITS and 28S seqences.

*Additional examined material*: COSTA RICA: Puntarenas, Coto Brus, Las Mellizas, La Amistad Lodge, near Parque internacional La Amistad, 7 June 2003, R.E. Halling 8361 (NY792792).

*Commentary*: Some of the morphological differences (more strongly zonate and viscose) mentioned in the diagnosis of this Costan Rican species may in part be due to the different climatic conditions inside the mountainous oak forests where it is found. However, there appears to exist a clear color difference between the two species and the very bright yellow tinges that characterize most *M*. *furcata* collections have not yet been observed in *M*. *mesoamericana*.

**2.3. *Multifurca orientalis*** X.H. Wang, Index Fungorum 358: 1. 2018. Fig 5H

*Index Fungorum number*: IF554375

*Specimens examined*: CHINA. Anhui Prov.: Qianshan Co., Tianzhu Mt., near Foguang Temple, elev. 630 m, N30°43'17.73'' E116°27'18.31'', under mixed forest of *Pinus taiwanensis* and fagaceous trees, 29 August 2011, X.H. Wang 3034 (holotype, HKAS 73577, KUN!). Guangdong Prov.: Fengkai Co., Heishiding, elev. 400 m, 12 September 2012, F. Li 1055 (HKAS 77779, KUN).

**2.4 *Multifurca pseudofurcata*** X.H. Wang, Index Fungorum 358: 1. 2018. Fig 5I

*Index Fungorum number*: IF554372

*Specimens examined*: CHINA. Guizhou Prov.: Kaiyang Co., Longgang, Gangzhai forest farm, elev. 1200–1300 m, under mixed forest of *Pinus massoniana* and fagaceous trees, 19 June 2010, X.H. Wang 2374 (HKAS 61341, KUN). Sichuan Prov.: Luzhou, Naxi, 8 October 2006, B. Xiao s.n. (HKAS 52928, KUN). Yunnan Prov.: Lancang Co., Qianmai, Longshan Village, elev. 950 m, under *Pinus kesiya* var. *langbianensis*, 18 July 2002, F.Q. Yu 775 (HKAS 41904, KUN); Lijiang Co., Jin’an, elev. 2600 m, N26°53'37.97'' E100°19'12.72'', under *P*. *yunnanensis*, 3 August 2011, Q. Cai 525 (HKAS 70121, KUN); Nanhua Co., roadside from Nanhua to Yaoan (provincial road 217), 15 km from Nanhua county town, Maan Mt., N25°17'43.50'' E101°16'18.14'', elev. 2200 m, 3 August 2009, G. Wu 85 (HKAS 57617, KUN), L.P. Tang 1093 (HKAS 57050, KUN); Mengla Co., Longmen, Nanman Mt., elev. 850–900 m, 27 September 1974, M. Zang 1868 (HKAS 1868, KUN); Puer Pref., Caiyanghe, 11 July 2014, J. Li 61 (HKAS 85885, KUN); ibid., 6 July 2000, M. Zang 13589 (HKAS 36369, KUN); Weixi Co., Qizong, roadside from Qizong to Tacheng, elev. 2011 m, N27°34'53.91'' E99°29'24.12'', under mixed forest of *Pinus yunnanensis* and *Quercus* trees, 19 September 2010, X.H. Wang 2844 (HKAS 62040, KUN); Weixi Co., Qizong., roadside from Qizong to Tacheng, elev. 1990 m, N27°34'53.48'' E99°29'51.69'', under mixed forest of *Pinus yunnanensis* and fagaceous trees, 14 October 2011, X.H. Wang 3205 (holotype, HKAS 75815, KUN!); Yunlong Co., Nuodeng town, Nuodeng village, elev. 1940 m, 26 August 2011, R. Wang 2011-yl-69 (HKAS 72886, KUN).

*Commentary*: This species was misidentified as *M*. *furctata* in China [19]. Unlike *M*. *furcata*, its latex does not change, or discolours dull yellowish-green or stains lamellae pale brownish. This is the only morphological difference we could found between the two species. There are roughly two groups based on DNA data (S2 Fig) but morphologically they cannot be separated.

**2.5 *Multifurca stenophylla*** (Berk.) T. Lebel, C.W. Dunk & T.W. May, Mycological Progress 12: 499 (2013). Fig 5J

*Basionym*: *Lactarius stenophyllus* Berk. in J.D. Hooker, Flora Tasmaniae 2: 248. 1859 (as ‘1860’).

≡ *Lactifluus stenophyllus* (Berk.) Kuntze, Revis. gen. Pl. II: 857. 1891.

*Examined material*: AUSTRALIA. Victoria: Eastern Highlands, Yarra State Forest, Big Creek Rd, Ada Tree Walk, J.E. Tonkin 1201, 25 Mar. 2005 (MEL2297389); Central Highlands, Noojee State Forest, Link Rd, C. Dunk CWD 584, 28 Feb. 2005 (MEL2361954); Great Otway National Park, Aire Crossing, Halls Ridge Rd, C. Dunk CWD 600, 20 Mar. 2005 (MEL2361973). Tasmania: Tarkine Wilderness, Great Western Hwy, Mt Donaldson Track, T. Lebel & P. Catcheside TL2462, 3 May 2012 (MEL 2362079); Tarkine Wilderness, Great Western Hwy, Mt Donaldson Track, T. Lebel 2335, 14 May 2010 (MEL2352529).

*Commentary*: This taxon, being described in the mid-nineteenth century (as “*Lactarius*”) is the ‘oldest’ species of *Multifurca*. It is found in habitat dominated by Myrtaceae (mainland Australia) or with scattered Nothofagaceae (Tasmania).

### Key to the species

1. Pileus surface indistinctly zonate to azonate, mostly whitish. No milk. Taste mild to mostly bitter or nauseous. Hymenophore initially mostly white, turning to yellowish orange with age. Spores more or less subreticulate. Lactifers ending in pseudocystidia not present or infrequent................................................ ***Multifurca* subg. *Multifurca*** 2

1. Pileus surface distinctly zonate, ochraceous or yellow. Milk white. Taste acrid. Hymenophore cream to yellow. Spores irregularly warty or with some very short crests, not subreticulate. Lactifer network ending in typical and very abundant pseudocystidia in lamellar trama and hymenium............. ***Multifurca* subg. *Furcata*** 6

2. Pileus and stipe ochraceous yellow to pale yellowish brown. Pseudocystidia present in hymenium. Hymenial gloeocystidia not well-developed, negligible. Under dipterocarps and fagaceous trees. Thailand and southern China................***M*. *zonaria***

2. Pileus and stipe whitish. Pseudocystidia absent. Hymenial gloeocystidia well-developed, emergent, originating from deep within the lamellar trama..................3

3. Small-sized (< 5 cm diam.). With distinct pileocystidia. Under *Nothofagus*. New Caledonia......................................................................***M*. *aurantiophylla***

3. Big-sized (> 5 cm diam.). Pileocystidia absent or doubtful. Under Myrtaceae, Pinaceae or Fagaceae.............................................................................4

4. Hymenial gloeocystidia of one type, never minutely mucronate, nor strongly inflated in the submerged part. Growing with Myrtaceae. Northeastern Australia................................................................................***M*. *australis***

4. Hymenial gloeocystidia of two types, one type frequently inflated in the submerged part. Growing with Pinaceae and Fagaceae....................................................5

5. Spores with subreticulate, low ornamentation. Penetrating smell of citronella. Under Pinaceae or Fagaceae. Southeastern USA.......................***M*. *ochricompacta***

5. Spores with slightly better developed ornamentation. Smell resinaceous. With *Pinus* or Fagaceae. Himalayan India and China.............................***M*. *roxburghiae***

6. Spores densely warty-crested. Under Nothofagaceae or Myrtaceae. Southern Australia.............................................................................***M*. *stenophylla***

6. Spores with low and fine warts, sometimes nearly invisible. Species associated with Pinaceae or Fagaceae............................................................................7

7. Asia. Cryptic..........................................***M*. *orientalis*** and ***M*. *pseudofurcata***

7. America .........................................................................................8

8. Poorly zonate, dry cap, bright yellow to orange yellow. Southeastern USA....................................................................................***M*. *furcata***

8. Distinctly zonate, viscose cap, dull pinkish to yellowish brown. Costa Rica...........................................................................***M*. *mesoamericana***

## Supporting information

S1 Fig Maximum Likelihood (ML) phylogram of *Multifurca*, *Lactarius*, *Lactifluus* and *Russula* generated by the 28S-*rpb2* combined dataset. The tree is rooted with midpoint. ML Bootstrap proportions higher than 70% are indicated above the branches.
(TIF)

S2 Fig Maximum Likelihood (ML) phylograms of *Multifurca* based on the ITS, 28S, *rpb2* and ITS-28S-*rpb2* combined datasets. Trees are rooted with *Lactarius pubescens*. Bootstrap proportions higher than 70% in the ML analysis (ML-BP) and Posterior probabilities of the Bayesian Inference (BI-PP) higher than 95% are indicated above and below the branches respectively or as ML-BP/BI-PP by the node. Thick black branches in the ITS-28S-*rpb2* tree represent nine of the ten terminal evolutionary lineages determined by Genealogical Concordance Phylogenetic Species Recognition. Green stars represent phylogenetic species. The four samples of *M*. *pseudofurcata* group 2 formed an evolutionary lineage and phylogenetic species, but did not form a monophyletic clade in the ITS-28S-*rpb2* tree.
(TIF)

S3 Fig Chronogram and estimated divergence times of *Multifurca* and other representatives of fungi. The chronogram was generated from biogeographic analysis using the 28S-*rpb2* data in BEAST. Ascomycota-Basidiomycota divergence time of 500–650 Ma was used calibration point. The geological time scale is in millions of years ago.
(TIF)

S1 Table Newly designed internal primers in this study. (DOCX)

S2 Table Species and samples used for divergence time estimation of *Multifurca* implemented in BEAST. (DOCX)

S1 Text Matrices of within-group and between-group genetic distances estimated using ITS and ITS-LSU-*rpb2* sequence data. (DOCX)
