# Supplementary figures and images for: Phylogeny, biogeography and taxonomic re-assessment of Multifurca (Russulaceae, Russulales) using three-locus data
Source: PLoS One. 2018 Nov 7;13(11):e0205840. doi: 10.1371/journal.pone.0205840 (PMC6221288; doi:10.1371/journal.pone.0205840)

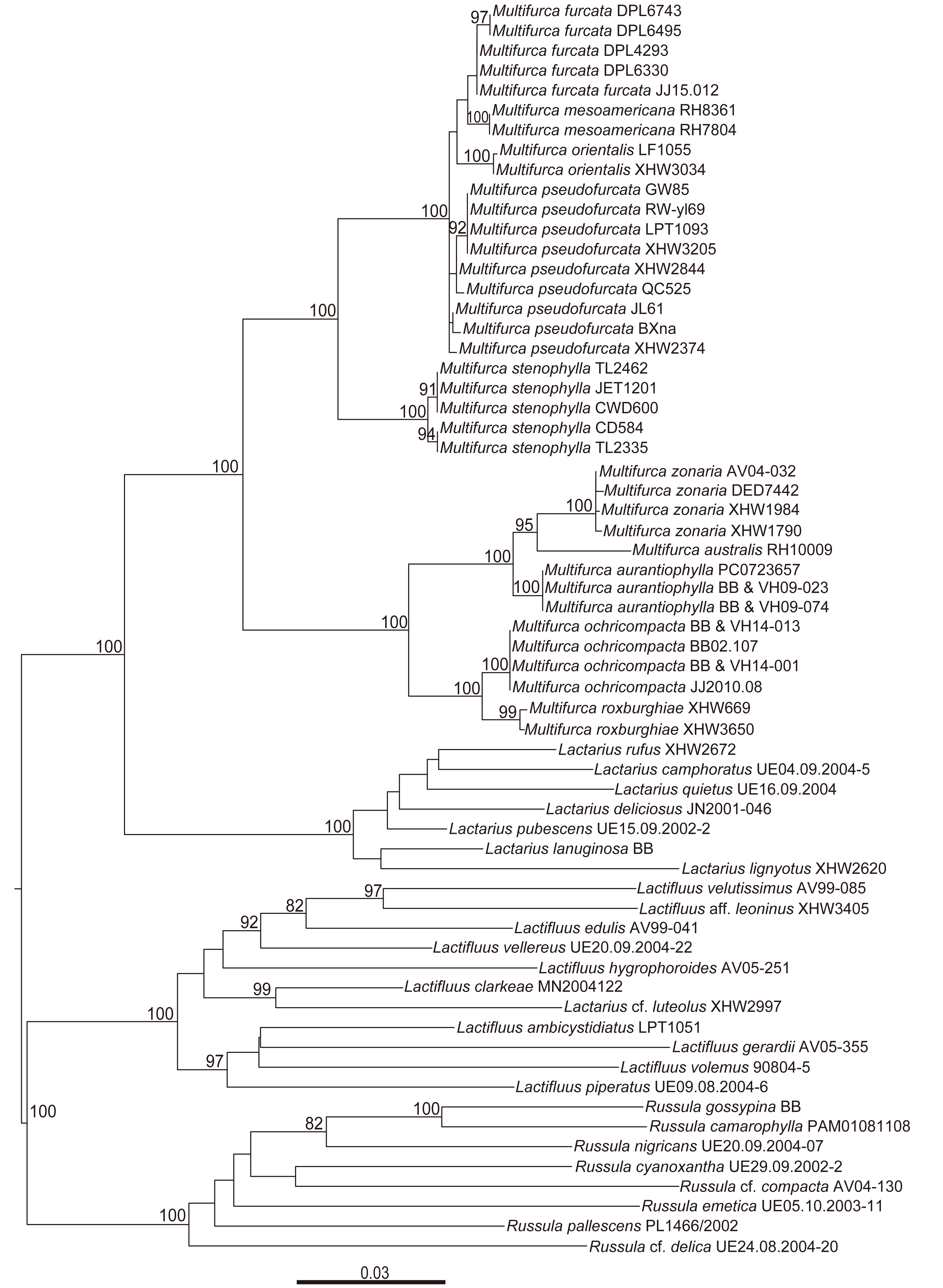

Supplement: S1 Fig — The tree is rooted with midpoint. ML Bootstrap proportions higher than 70% are indicated above the branches. (TIF) [file pone.0205840.s001.tif]

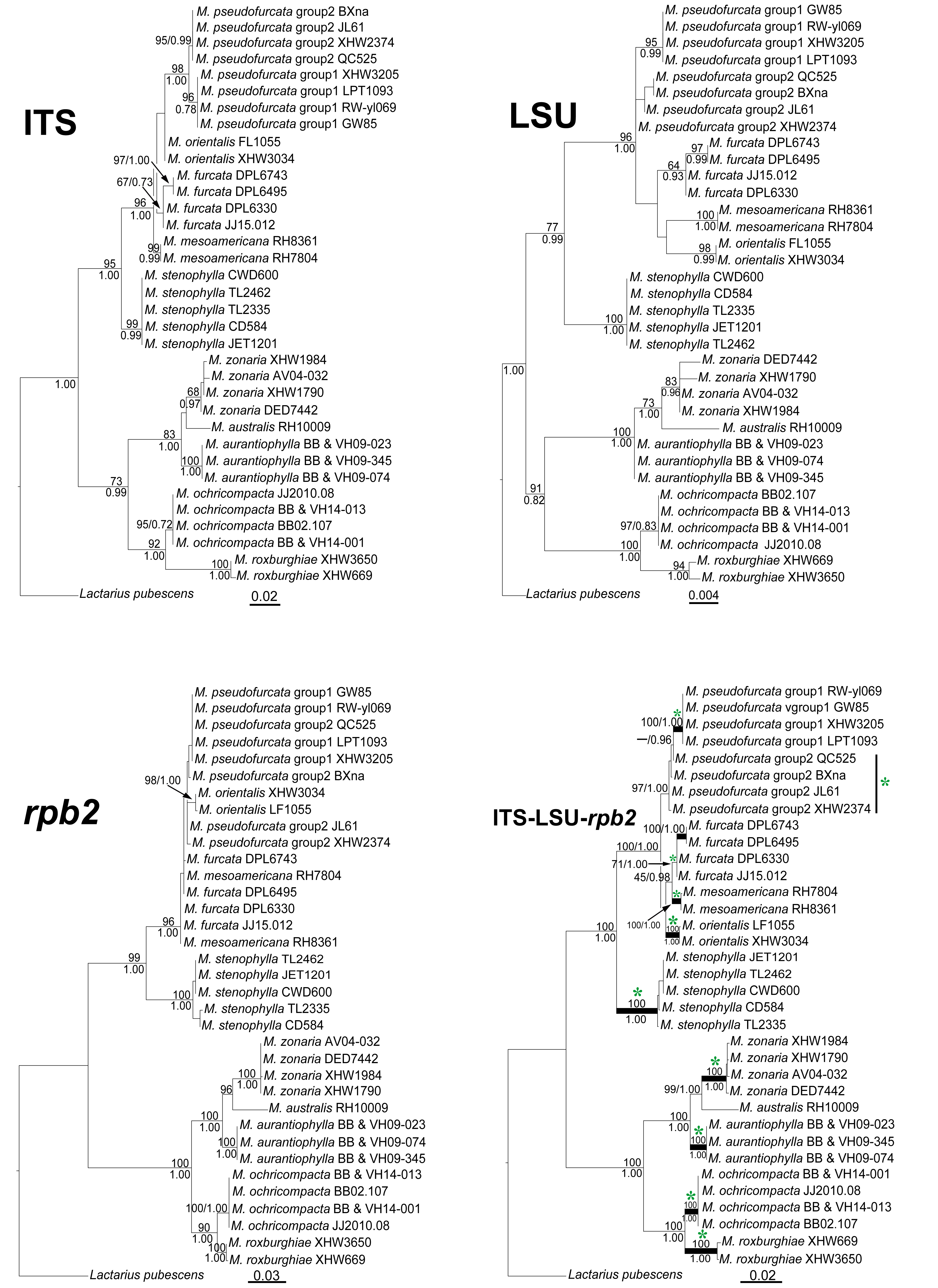

Supplement: S2 Fig — Trees are rooted with Lactarius pubescens. Bootstrap proportions higher than 70% in the ML analysis (ML-BP) and Posterior probabilities of the Bayesian Inference (BI-PP) higher than 95% are indicated above and below the branches respectively or as ML-BP/BI-PP by the node. Thick black branches in the ITS-28S-rpb2 tree represent nine of the ten terminal evolutionary lineages determined by Genealogical Concordance Phylogenetic Species Recognition. Green stars represent phylogenetic species. The four samples of M. pseudofurcata group 2 formed an evolutionary lineage and phylogenetic species, but did not form a monophyletic clade in the ITS-28S-rpb2 tree. (TIF) [file pone.0205840.s002.tif]

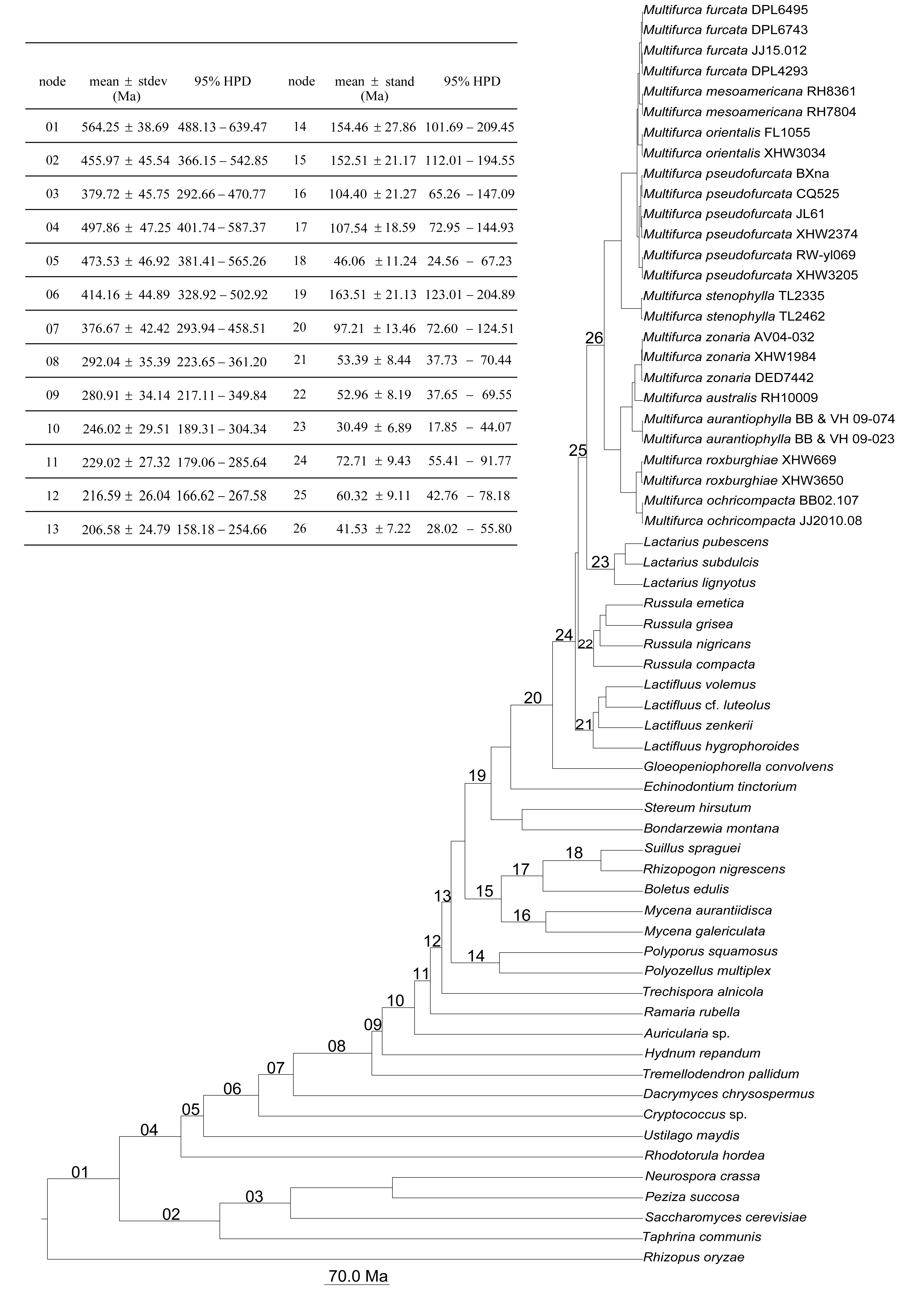

Supplement: S3 Fig — The chronogram was generated from biogeographic analysis using the 28S-rpb2 data in BEAST. Ascomycota-Basidiomycota divergence time of 500–650 Ma was used calibration point. The geological time scale is in millions of years ago. (TIF) [file pone.0205840.s003.tif]
