# Supplementary material for: Phylogeny, biogeography and taxonomic re-assessment of Multifurca (Russulaceae, Russulales) using three-locus data
Source: PLoS One. 2018 Nov 7;13(11):e0205840. doi: 10.1371/journal.pone.0205840 (PMC6221288; doi:10.1371/journal.pone.0205840)
Supplement: S1 Table — (DOCX) [file pone.0205840.s004.docx]

| **primer** | **sequences (5’− 3’)** | **counterpart primer** | **locus (gene)** | **target taxon** |
| --- | --- | --- | --- | --- |
| ITS-MMF1 | GTGCATCACCRCGYRGGC | 5.8S-new or ITS2 | Part of ITS1 | *Multifurca* |
| ITS-MMR1 | GCCYRCGYGGTGATGCAC | ITS1F or ITS5 | Part of ITS1 | *Multifurca* |
| ITS-MMF2 | TYGACGTGATAAGATSTTT | ITS4 | Part of ITS2 | *Multifurca* |
| ITS-MMR2 | AAASATCTTATCACGTCRA | ITS3 or 5.8SR | Part of ITS2 | *Multifurca* |
| 5.8S-new | TCGGAATACCARGGGGYGCAAGG | ITS-MMF1 | Part of ITS1 | *Multifurca* |
| FurcataIF1 | ATCCATCTCACCCCTTGTGCATC | 5.8S-new or ITS2 | Part of ITS1 | *M. furcata* complex |
| FurcataIR1 | TGGACCRGTCTGATCCAGAG | ITS1F or ITS5 | Part of ITS1 | *M.* subg. *Furcata* |
| FurcataIF2 | TCCTCTCAAATGGATTAGTGG | ITS4 | Part of ITS2 | *M.* subg. *Furcata* |
| FurcataIR2 | CGTAGAAAGATCTTATCACGTC | 5.8S-new or ITS2 | Part of ITS2 | *M.* subg. *Furcata* |
| Multi-LF | CCGATAGCGAACAAGTACCGTGA | LR3 | Part of LSU1 | *Multifurca* |
| Multi-LR | TTCACGTACTGTTTCACTCTC | LROR | Part of LSU1 | *Multifurca* |
| RoxLF1 | TCATAGAGGGTGAGAATCCCGT | RoxLR2 | Part of LSU | *Multifurca* |
| RoxLR1 | ACGGGATTCTCACCCTCTATGA | LROR | Part of LSU | *Multifurca* |
| RoxLF2 | CGGGTCAGCATCAATTTTGCC | RoxLR3 | Part of LSU | *Multifurca* but *M. ochricompacta* |
| RoxLR2 | AAATTGATGCTGACCCGTC | RoxLF1 | Part of LSU | *Multifurca* but *M. ochricompacta* |
| RoxLF3 | CTATGCCTGAATAGGGTGA | LR5 | Part of LSU | *Multifurca* |
| RoxLR3 | TCACCCTATTCAGGCATAG | ROXLF2 | Part of LSU | *Multifurca* |
| Mul-RF1 | TTCATCGACGCTCACTACGA | RoxRF2 | Part of rpb2 | *Multifurca* |
| Mul-RR1 | TCGTAGTGAGCGTCGATGAA | 6F | Part of rpb2 | *Multifurca* |
| Mul-RF2 | TGCGCGACATTCGGGAAAGA | RoxRR3 | Part of rpb2 | *M.* subg. *Multifurca* |
| Mul-RR2 | TCTTTCCCGAATGTCGCGCA | RoxRF1 | Part of rpb2 | *M.* subg. *Multifurca* |
| Mul-RF3 | TGATCTGCATGACCCCAGAAGA | RoxRR4 | Part of rpb2 | *M.* subg. *Multifurca* |
| Mul-RR3 | TCTTCTGGGGTCATGCAGATCA | RoxRF2 | Part of rpb2 | *M.* subg. *Multifurca* |
| Mul-RF4 | GATCTTGGGTATCTGCGCC | 7CR | Part of rpb2 | *M.* subg. *Multifurca* |
| Mul-RR4 | GGCGCAGATACCCAAGATC | RoxRF3 | Part of rpb2 | *M.* subg. *Multifurca* |
| MMF-2 | TGGCTCAATCAGGGTASYACGGA | 7CR | Part of rpb2 | *Multifurca* |
| MMR-2 | TCATGCAGATCATMACTGTTTCC | 6F | Part of rpb2 | *Multifurca* |
| FurcataRR1 | AATGGAGACCTCTGGGTGGACA | 6F | Part of rpb2 | *M. furcata* complex |
| FurcataRF1 | CCGACCAATCTCATTGAGACTCT | 7CR | Part of rpb2 | *M. furcata* complex |

Newly designed internal primers in this study.

Schematic map of the newly designed primers (in bold) in the alignments of ITS, LSU and *rpb2*

**
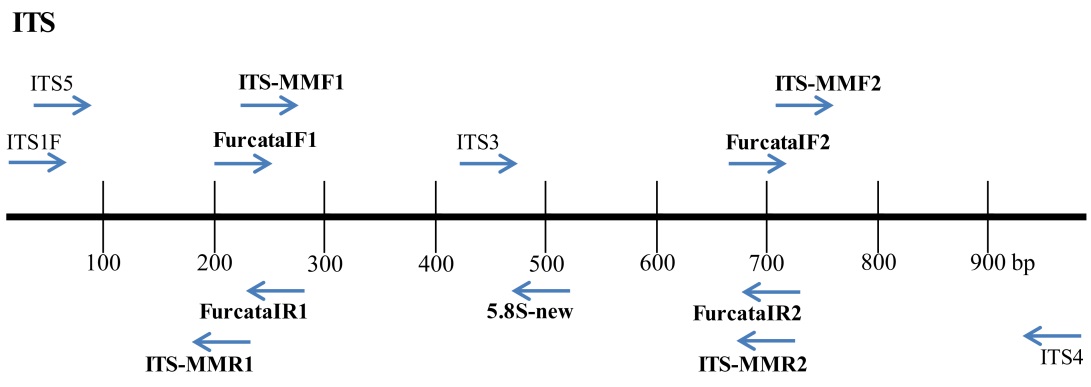
**

*
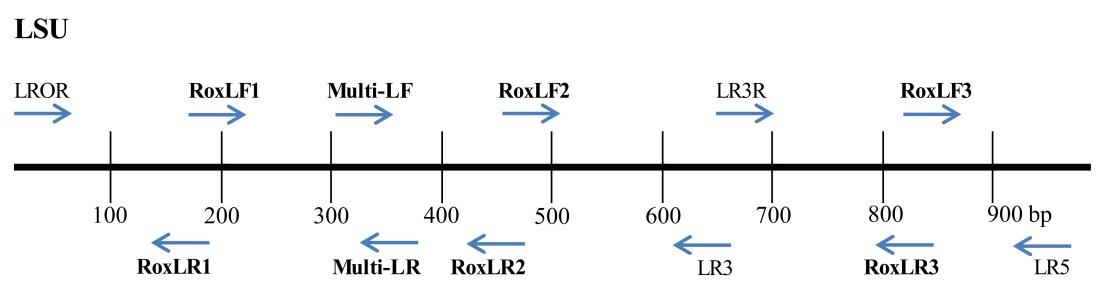
*

**
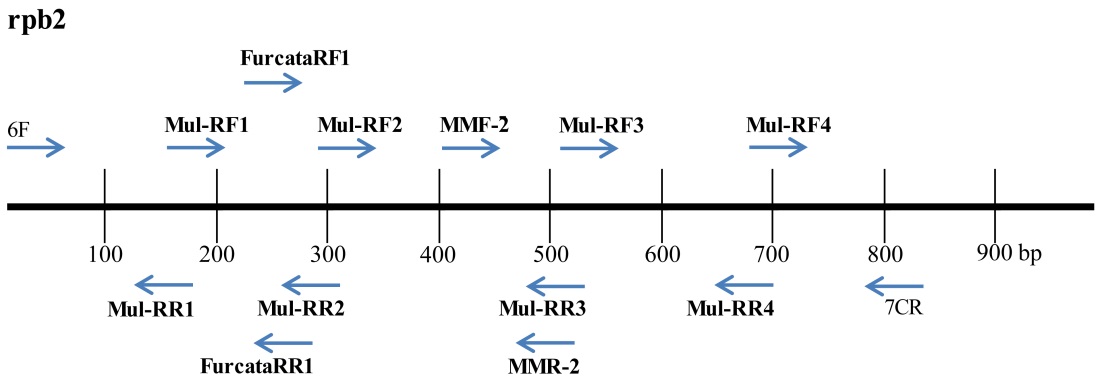
**
