# Supplementary material for: Phylogeny, biogeography and taxonomic re-assessment of Multifurca (Russulaceae, Russulales) using three-locus data
Source: PLoS One. 2018 Nov 7;13(11):e0205840. doi: 10.1371/journal.pone.0205840 (PMC6221288; doi:10.1371/journal.pone.0205840)
Supplement: S2 Table — (DOCX) [file pone.0205840.s005.docx]

Species and samples used for divergence time estimation of *Multifurca* implemented in BEAST.

| **Species** | **Sample ID (herbarium)** | **28S** | ***rpb2*** | **Phylogenetic position** |
| --- | --- | --- | --- | --- |
| *Multifurca aurantiophylla* | BB & VH 09-023 (PC) | MH063839 | MH061171 | Basidiomycota-russuloid clade |
| *Multifurca aurantiophylla* | BB & VH 09-074 (PC) | MH063837 | MH061169 | Basidiomycota-russuloid clade |
| *Multifurca australis* | R.E. Halling 10009 (BRI) | MH063836 | MH061168 | Basidiomycota-russuloid clade |
| *Multifurca furcata* | J. Justice 15.012 (PC) | MH063827 | MH061156 | Basidiomycota-russuloid clade |
| *Multifurca furcata* | D.P. Lewis 4293 (F) | MH063828 | MH061157 | Basidiomycota-russuloid clade |
| *Multifurca furcata* | D.P. Lewis 6495 (F) | MH063831 | MH061160 | Basidiomycota-russuloid clade |
| *Multifurca furcata* | D.P. Lewis 6743 (F) | MH063830 | MH061159 | Basidiomycota-russuloid clade |
| *Multifurca mesoamericana* | R. Halling 7804 (NY) | DQ421994 | DQ421927 | Basidiomycota-russuloid clade |
| *Multifurca mesoamericana* | R. Halling 8361 (NY) | DQ421995 | DQ421928 | Basidiomycota-russuloid clade |
| *Multifurca ochricompacta* | B. Buyck 02.107 (PC) | DQ421984 | DQ421940 | Basidiomycota-russuloid clade |
| *Multifurca ochricompacta* | J. Justice 2010.08 (PC) | MH063844 | MH061176 | Basidiomycota-russuloid clade |
| *Multifurca orientalis* | X.H. Wang 3034 (KUN) | MH063825 | MH061154 | Basidiomycota-russuloid clade |
| *Multifurca orientalis* | F. Li 1055 (KUN) | MH063826 | MH061155 | Basidiomycota-russuloid clade |
| *Multifurca pseudofurcata* | Q. Cai 525 (KUN) | MH063824 | MH061153 | Basidiomycota-russuloid clade |
| *Multifurca pseudofurcata* | J. Li 61 (KUN) | MH063822 | MH061151 | Basidiomycota-russuloid clade |
| *Multifurca pseudofurcata* | R. Wang yl-69 (KUN) | MH063818 | MH061147 | Basidiomycota-russuloid clade |
| *Multifurca pseudofurcata* | X.H. Wang 2374 (KUN) | MH063821 | MH061150 | Basidiomycota-russuloid clade |
| *Multifurca pseudofurcata* | X.H. Wang 3205 (KUN) | MH063819 | MH061148 | Basidiomycota-russuloid clade |
| *Multifurca pseudofurcata* | B. Xu s.n. (HKAS52928, KUN) | MH063823 | MH061152 | Basidiomycota-russuloid clade |
| *Multifurca roxburghiae* | X.H. Wang 669 (KUN) | MH063841 | MH061173 | Basidiomycota-russuloid clade |
| *Multifurca roxburghiae* | X.H. Wang 3650 (KUN) | MH063840 | MH061172 | Basidiomycota-russuloid clade |
| *Multifurca stenophylla* | T. Lebel 2335 (MEL) | JX266636 | MH061164 | Basidiomycota-russuloid clade |
| *Multifurca stenophylla* | T. Lebel & P. Catcheside TL2462 (MEL) | MH063832 | MH061161 | Basidiomycota-russuloid clade |
| *Multifurca zonaria* | D.E. Desjardin 7442 (SFSU, PC) | DQ421990 | DQ421942 | Basidiomycota-russuloid clade |
| *Multifurca zonaria* | A. Verbeken 2004-032 (GENT) | DQ422000 | DQ421947 | Basidiomycota-russuloid clade |
| *Multifurca zonaria* | X.H. Wang 1984 (KUN) | MH063834 | MH061166 | Basidiomycota-russuloid clade |
| *Auricularia* sp. | AFTOL-676 (PBM2295) | DQ366278 | AY634277 | Basiciomycota-Auriculales |
| *Boletus edulis* | HMJAU4637 (HMJAU) | KF112455 | KF112704 | Basidiomycota-boletoid clade |
| *Bondarzewia montana* | AFTOL 452 (DAOM415) | DQ234539 | AY218474 | Basidiomycota-russuloid clade |
| *Cryptococcus* sp*.* | AFTOL 719 (CBS 681.93) | AY646103 | DQ408137 | Basidiomycota-Tremellomycetidae |
| *Dacrymyces chrysospermus* | FPL11353 (not specified) | AF287855 | AY218480 | Basidiomycota-Dacrymycetales |
| *Gloeopeniophorella convolvens* | OM19405 (not specified) | Genome data | Genome data | Basidiomycota-russuloid clade |
| *Echinodontium tinctorium* | AFTOL ID455 (DAOM16666) | AF393056 | AY218482 | Basidiomycota-russuloid clade |
| *Hydnum repandum* | BB07.341 (PC) | KF294643 | KF294720 | Basidiomycota-cantharelloid |
| *Lactarius lignoytus* | U. Eberhardt 06.09.2003-5 (UPS) | DQ422993 | DQ421926 | Basidiomycota-russuloid clade |
| *Lactarius pubescens* | U. Eberhardt 15.09.2002-2 (UPS) | DQ421996 | DQ421929 | Basidiomycota-russuloid clade |
| *Lactarius subdulcis* | JV2006-024 (GENT) | KF133312 | KF | Basidiomycota-russuloid clade |
| *Lactifluus* cf. *luteolus* | X.H. Wang 2997 (KUN) | KC154124 | KC154150 | Basidiomycota-russuloid clade |
| *Lactifluus hygrophoroides* | A. Verbeken 05-251 (GENT) | HQ318208 | HQ328936 | Basidiomycota-russuloid clade |
| *Lactifluus volemus* | 90804-5 (GENT) | JN389010 | JN375612 | Basidiomycota-russuloid clade |
| *Lactifluus zenkeri* | A. Verbeken 11-050 (GENT) | KR364182 | KR364297 | Basidiomycota-russuloid clade |
| *Mycena aurantiidisca* | AFTOL-ID 1685 (WTU) | DQ470811 | DQ474122 | Basidiomycota-euagarics |
| *Mycena galericulata* | AFTOL-ID 727 | AY647216 | DQ385888 | Basidiomycota-euagarics |
| *Neurospora crassa* | OR74A (not specified) | Genome data | XM_952013 | Ascomycota-Pezizomycotina |
| *Peziza succosa* | KH-98-07 (C) | AF335166 | AY500487 | Ascomycota-Pezizomycotina |
| *Polyozellus multiplex* | AFTOL-ID 677 | AY634275 | DQ408134 | Basidiomycota-thelephoroid clade |
| *Polyporus squamosus* | AFTOL-ID704 | AY629320 | DQ408120 | Basidiomycota-polyporoid clade |
| *Ramaria rubella* | AFTOL-ID 724 | AY645057 | AY786064 | Basidiomycota-gomphoid clade |
| *Rhizopogon nigrescens* | MB06-070 | GU187594 | GU187806 | Basidiomycota-boletoid clade |
| *Rhizopus oryzae* | 99-880 (not specified) | Genome data | Genome data | Zygomycota |
| *Rhodotorula hordea* | AFTOL-ID 674 (CBS) | AY631901 | DQ234555 | Basidiomycota-Pucciniomycotina |
| *Russula compacta* | B. Buyck 06.295 (PC) | KU237480 | KU237766 | Basidiomycota-russuloid clade |
| *Russula emetica* | U. Eberhardt 05.10.2003-11 (UPS) | DQ421997 | DQ421943 | Basidiomycota-russuloid clade |
| *Russula grisea* | B. Buyck 07.184 (PC) | KU237509 | KU237795 | Basidiomycota-russuloid clade |
| *Russula nigricans* | U. Eberhardt 20.09.2004-07 (UPS) | DQ422010 | DQ421952 | Basidiomycota-russuloid clade |
| *Saccharomyces cerevisiae* | S288C for rpb2, 28S not specified | J01355 | M15693 | Ascomycota-Saccharomycotina |
| *Stereum hirtutum* | AFTOL-ID 492 (FPL 8805) | AF393078 | AY218520 | Basidiomycota-russuloid clade |
| *Suillus spraguei* | AFTOL-ID 717 | AY684154 | AY786066 | Basidiomycota-boletoid clade |
| *Taphrina communis* | NRRL T-755 | AY640972 | AY641083 | Ascomycota-Taphrinomycotina |
| *Trechispora alnicola* | AFTOL-ID 665 (CBS577.83) | DQ408135 | AY635768 | Basidiomycta-Trechisporoid clade |
| *Tremellodendron pallidum* | AFTOL 699 (PBM2324) | AY745701 | DQ408132 | Basidiomycota-Sebacinales |
| *Ustilago maydis* | CBS 504.76 (CBS) | AF453938 (MS115) | KP323090 | Basidiomycota-Ustilaginomycetes |
