# Supplementary material for: Phylogeny, biogeography and taxonomic re-assessment of Multifurca (Russulaceae, Russulales) using three-locus data
Source: PLoS One. 2018 Nov 7;13(11):e0205840. doi: 10.1371/journal.pone.0205840 (PMC6221288; doi:10.1371/journal.pone.0205840)
Supplement: S1 Text — (DOCX) [file pone.0205840.s006.docx]

**Within-group genetic distances estimated using ITS sequence data**

Phylogenetic species recognized Distance estimated (D) Standard error (SE)

[ 1] M. pseudofurcata group 1 0.000 0.000

[ 2] M. pseudofurcata group 2 0.004 0.001

[ 3] M. orientalis 0.000 0.000

[ 4] M. mesoamericana 0.000 0.000

[ 5] M. furcata 0.002 0.001

[ 6] M. sp (sample 2676) n/c n/c

[ 7] M. stenophylla 0.000 0.000

[ 8] M. zonaria 0.004 0.002

[ 9] M. australis (sample RH10009) n/c n/c

[10] M. aurantiophylla **0.005 0.002**

[11] M. roxburghiae 0.003 0.002

[12] M. ochricompacta 0.001 0.001

**Between-group genetic distances estimated using ITS sequence data**

| SE  D | [1] | [2] | [3] | [4] | [5] | [6] | [7] | [8] | [9] | [10] | [11] | [12] |
| --- | --- | --- | --- | --- | --- | --- | --- | --- | --- | --- | --- | --- |
| [ 1] |  | 0.003 | 0.006 | 0.007 | 0.006 | 0.008 | 0.011 | 0.021 | 0.025 | 0.019 | 0.019 | 0.018 |
| [ 2] | 0.009 |  | 0.006 | 0.007 | 0.005 | 0.007 | 0.010 | 0.021 | 0.024 | 0.019 | 0.019 | 0.017 |
| [ 3] | 0.024 | 0.022 |  | 0.006 | 0.005 | 0.008 | 0.010 | 0.021 | 0.025 | 0.019 | 0.019 | 0.017 |
| [ 4] | 0.036 | 0.033 | 0.022 |  | 0.004 | **0.006** | 0.010 | 0.020 | 0.023 | 0.018 | 0.019 | 0.017 |
| [ 5] | 0.026 | 0.022 | 0.018 | 0.013 |  | 0.006 | 0.009 | 0.020 | 0.024 | 0.019 | 0.019 | 0.017 |
| [ 6] | 0.037 | 0.032 | 0.034 | **0.022** | 0.026 |  | 0.011 | 0.020 | 0.024 | 0.018 | 0.020 | 0.018 |
| [ 7] | 0.062 | 0.057 | 0.054 | 0.054 | 0.051 | 0.060 |  | 0.018 | 0.021 | 0.018 | 0.018 | 0.016 |
| [ 8] | 0.164 | 0.162 | 0.159 | 0.151 | 0.153 | 0.151 | 0.146 |  | 0.010 | 0.009 | 0.015 | 0.013 |
| [ 9] | 0.192 | 0.191 | 0.180 | 0.183 | 0.188 | 0.178 | 0.171 | 0.057 |  | 0.010 | 0.018 | 0.016 |
| [10] | 0.145 | 0.144 | 0.136 | 0.140 | 0.145 | 0.132 | 0.138 | 0.054 | 0.058 |  | 0.015 | 0.013 |
| [11] | 0.153 | 0.152 | 0.154 | 0.161 | 0.157 | 0.166 | 0.141 | 0.122 | 0.143 | 0.121 |  | 0.009 |
| [12] | 0.136 | 0.135 | 0.131 | 0.137 | 0.135 | 0.141 | 0.117 | 0.097 | 0.115 | 0.093 | 0.055 |  |

**Within-group genetic distances estimated using ITS-LSU-*rpb*2 sequence data**

Phylogenetic species recognized D SE

[ 1] M. pseudofurcata group 1 0.000 0.000

[ 2] M. pseudofurcata group 2 **0.003 0.001**

[ 3] M. orientalis 0.000 0.000

[ 4] M. mesoamericana 0.000 0.000

[ 5] M. furcata 0.002 0.001

[ 6] M. stenophylla 0.001 0.000

[ 7] M. zonaria 0.002 0.001

[ 8] M. australis (sample RH10009) n/c n/c

[ 9] M. aurantiophylla 0.000 0.000

[10]M. roxburghiae 0.002 0.001

[11] M. ochricompacta 0.000 0.000

**Between-group genetic distances estimated using ITS-LSU-*rpb2* sequence data**

| SE  D | [1] | [2] | [3] | [4] | [5] | [6] | [7] | [8] | [9] | [10] | [11] |
| --- | --- | --- | --- | --- | --- | --- | --- | --- | --- | --- | --- |
| [ 1] |  | 0.001 | 0.003 | 0.003 | 0.002 | 0.006 | 0.010 | 0.010 | 0.009 | 0.010 | 0.009 |
| [ 2] | 0.004 |  | 0.002 | 0.003 | 0.002 | 0.006 | 0.010 | 0.010 | 0.009 | 0.010 | 0.009 |
| [ 3] | 0.014 | 0.012 |  | 0.002 | 0.002 | 0.005 | 0.010 | 0.010 | 0.009 | 0.010 | 0.009 |
| [ 4] | 0.020 | 0.015 | 0.015 |  | 0.002 | 0.005 | 0.010 | 0.010 | 0.009 | 0.010 | 0.010 |
| [ 5] | 0.014 | 0.010 | 0.012 | 0.008 |  | 0.005 | 0.010 | 0.011 | 0.009 | 0.010 | 0.009 |
| [ 6] | 0.048 | 0.045 | 0.049 | 0.049 | 0.044 |  | 0.009 | 0.010 | 0.000 | 0.009 | 0.008 |
| [ 7] | 0.119 | 0.115 | 0.121 | 0.118 | 0.113 | 0.111 |  | **0.005** | 0.004 | 0.008 | 0.008 |
| [ 8] | 0.126 | 0.123 | 0.126 | 0.126 | 0.124 | 0.122 | **0.037** |  | 0.004 | 0.008 | 0.008 |
| [ 9] | 0.108 | 0.105 | 0.108 | 0.109 | 0.108 | 0.102 | 0.030 | 0.037 |  | 0.007 | 0.007 |
| [10] | 0.110 | 0.107 | 0.112 | 0.113 | 0.108 | 0.100 | 0.075 | 0.086 | 0.069 |  | 0.004 |
| [11] | 0.102 | 0.099 | 0.103 | 0.105 | 0.101 | 0.093 | 0.067 | 0.077 | 0.060 | 0.026 |  |
